# A Review of Pathogen Transmission at the Backyard Chicken–Wild Bird Interface

**DOI:** 10.3389/fvets.2020.539925

**Published:** 2020-09-24

**Authors:** Andrea J. Ayala, Michael J. Yabsley, Sonia M. Hernandez

**Affiliations:** ^1^Department of Population Health, College of Veterinary Medicine, University of Georgia, Athens, GA, United States; ^2^Daniel B. Warnell School of Forestry and Natural Resources, University of Georgia, Athens, GA, United States; ^3^Southeastern Cooperative Wildlife Disease Study, Athens, GA, United States

**Keywords:** backyard chickens, wild birds, pathogen transmission, wildlife-livestock interface, emerging disease

## Abstract

Habitat conversion and the expansion of domesticated, invasive species into native habitats are increasingly recognized as drivers of pathogen emergence at the agricultural–wildlife interface. Poultry agriculture is one of the largest subsets of this interface, and pathogen spillover events between backyard chickens and wild birds are becoming more commonly reported. Native wild bird species are under numerous anthropogenic pressures, but the risks of pathogen spillover from domestic chickens have been historically underappreciated as a threat to wild birds. Now that the backyard chicken industry is one of the fastest growing industries in the world, it is imperative that the principles of biosecurity, specifically bioexclusion and biocontainment, are legislated and implemented. We reviewed the literature on spillover events of pathogens historically associated with poultry into wild birds. We also reviewed the reasons for biosecurity failures in backyard flocks that lead to those spillover events and provide recommendations for current and future backyard flock owners.

## Introduction

Transboundary emerging and reemerging infectious diseases are now increasingly recognized as interconnected threats to public health, livestock, and wildlife communities (1–3). In North America, almost 80% of World Organization for Animal Health (OIE) reportable pathogens require at least one wildlife species to complete their life cycles, and half of those are zoonotic (4). Almost two decades ago, Dobson and Foufopoulos (5) defined emergent pathogens as those whose “geographical range, host range, and/or prevalence” are expanding.

The livestock–wildlife interface is a landscape now associated with rising incidences and expanding distributions of OIE-reportable pathogens (6–9). The convergence of food animal production activities with wildlife habitats forms optimal circumstances for multihost pathogen and zoonosis emergence (10–13). A classic example of this phenomenon is *Mycobacterium bovis* in the United Kingdom and the Republic of Ireland, whereby cattle and wild European Badgers (*Meles meles*) remain locked in a spillover and spillback transmission loop that remains unsolved with contemporary disease control measures (14–16). Fundamentally, pathogen emergence at the livestock–wildlife interface is a consequence of simultaneous perturbations such as pathogen pollution, climate change, biodiversity loss, habitat fragmentation, and agricultural sprawl (17–23).

At a community level, localized biodiversity loss is a consequence of land-use change, which may result in a disproportionate number of competent or amplifying hosts (17, 24). These processes are ultimately shaped through the interactions of the most significant components of the interface: first, the stability of the wildlife community (25), second, the type of domestic animal production (26, 27), and finally, the level of ecosystem fragmentation (28, 29).

In short, pathogen emergence at the livestock–wildlife interface is the result of *any one or many* mechanisms. Nonetheless, those factors generally fall into three broad categories: changes among reservoir host and recipient host densities (6), the rate of habitat transformation (30), and the presence of multihost pathogens (31). Although one category (host, habitat, or pathogen) may appear to dominate in a given scenario, understanding how infectious diseases emerge from the livestock–wildlife interface requires a holistic approach of the host–habitat–pathogen triad (32–34).

### Poultry Agriculture and Wild Birds

The pathogen dynamics of poultry agriculture are unique among animal-based farming systems (35–38). Numerous bird species are commonly owned as pets as well as for food and are prominent in even the most developed environments (39, 40), have a unique physiology (41–43), and have an extraordinary taxonomic diversity (44–46). Many wild bird habitats also now suffer consistent encroachment or habitat conversion from the expansion of the poultry industry, which introduces new microbes or parasites into the wild bird community (47–50). In 2018, Birdlife International listed agricultural expansion and intensification as a major threat to 74% of the world's 1,469 globally threatened bird species (51). Global threats also include the introduction of invasive species, habitat conversion, pathogens, and logging for small-scale subsistence farming (51). As early as the mid-twentieth century, emerging and reemerging infectious diseases were increasingly recognized as threats to avian populations around the globe (52–54).

Historically, avian infectious diseases were not appreciated for their ability to influence populations and were relatively neglected for their part in causing species declines (55, 56). However, Sax et al. (57) tabulated 18 species of primarily endemic island species that were declared extinct, or extinct in the wild, due to infectious diseases coupled with invasive species displacement (57, 58) (Supplemental Table 1). An additional 77 wild bird species are currently at risk by the International Union for the Conservation of Nature (IUCN) and Birdlife International due to infectious diseases (51, 59) (Supplemental Table 1).

It is only recently that the mechanisms contributing to disease emergence in free-living birds have been investigated (60). In particular, endangered species and the role that avian diseases present in regulating their populations have garnered specific attention from conservationists and wildlife disease ecologists and epidemiologists (58, 61–69). Pathogens have been implicated in more than just mortality events. They have also exhibited sublethal population-level consequences to native birds such as fluctuations in breeding success and reductions to fecundity (70, 71). Pathogens have also been demonstrated to inhibit territorial defense mechanisms and allow non-native species to outcompete native birds from their habitats (70–73). Unfortunately, public perception also plays a role on the effects that pathogens have on avian populations. For example, in Southeast Asia, the preemptive culling of migratory waterfowl and shorebirds was sporadically used to control the spread of H5N1 (74, 75).

The spillover of poultry pathogens are reported regularly (76–79); unfortunately, much remains unknown regarding which wild species are consistently affected by which pathogen and the frequency with which these infections occur. The intensive practices that allow the poultry industry to produce more chickens [e.g., high-density rearing], also result in the maintenance of pathogens and increased pathogen transmission (36, 80). Poultry vaccines have contributed to the efficient, high-density, and high-output avian production that comprises the commercial poultry industry (81–83). However, the same vaccines may also provide optimal conditions for pathogens to spread rapidly due to a lack of sterilizing immunity (82, 84–89). In commercial systems, producers routinely apply live, killed, or vector-based vaccines to counter high-profile viruses and reduce economic losses (81).

However, high-density commercialization is not the only form of poultry production nor is it likely the most dominant worldwide. Backyard poultry remains the primary source of protein among many industrializing nations (90–94), while urban backyard chickens and keeping pet chickens are expanding among industrialized nations (95–97). In fact, in some European nations, the production of meat and eggs from backyard chickens are even outcompeting commercial industries (98). Overall, poultry production has increased over the last 70 years, and the growth of the industry is unlikely to slow in the foreseeable future (93, 99, 100). For the purpose of this review, we define backyard chickens as low densities of chickens that are owned by private individuals, which are not constricted by the biosecurity regulations common to commercial operations.

The recent rise in backyard poultry ownership or “microlivestock” among developed nations such as the United States is a unique subsection of the agricultural–wildlife interface, with tremendous implications for multispecies pathogen transmission (101–111). Common avian pathogens that have been isolated from, or that have been detected as, producing a previous infection through antibody testing from backyard chickens and turkeys around the globe include infectious bronchitis virus (IBV) (102, 112), Marek's disease virus (113), infectious bursal disease virus (IBDV) (102, 112, 113), *Mycoplasma* spp. (102, 112, 113), Newcastle disease virus (NDV) (102, 112, 114), *Escherichia coli* (113), and *Salmonella* spp. (101, 102, 113).

A meta-analysis performed by Wiethoelter et al. (8) reported that the free-living bird–poultry interface was the most highly reviewed interface in relation to the worldwide risks of emergent pathogens—primarily concerning highly pathogenic avian influenza virus (HPAIV). This increased scrutiny is partly due to the evidence supporting that backyard chickens are now being commonly kept within the world's largest cities (109, 115–117) to the edges of protected areas in developing nations (118–121)—suggesting that few terrestrial habitats have remained untouched and without risk from pathogens originating from poultry.

The explosive growth of backyard chickens as an industry results from increasing consumer demands for organic, humane meat and eggs (122–124), and the growing desire for a sustainable, farm-to-table food source (95). Also, many backyard chickens are kept as pets, sometimes resultant from humanely rehoming “spent” or nonlaying hens (125, 126). Last, in many areas, there remains a desire to continue long-standing cultural practices of maintaining family flocks (103–105). What remains unresolved are the potential long-term effects of the backyard poultry industry's unregulated biosecurity and limited veterinary care (127, 128) on native wild bird species. Poor pathogen management, coupled with the overlap of native wild bird habitats, serves to bring together the microbial communities endemic to domestic chickens (129, 130) and native birds (131, 132).

### The Backyard Chicken–Wild Bird Interface

At this time, the backyard chicken–wild bird interface currently lacks a unified, conceptual definition. Defining it is challenging, considering that local wild bird communities vary across geographic locations and that the husbandry protocols and regulations for backyard chickens differ from village to municipality. Moreover, wild birds differ in their susceptibility to “poultry” pathogens, and not all chicken breeds are susceptible to wild bird pathogens. Not surprisingly, reports of the backyard chicken–wild bird interface also take on many forms. In Egypt, a study of cocirculating LPAIV and HPAIV viruses in backyard chickens described the backyard–chicken wild bird interface as the zone where waterfowl and shorebirds interact with household chickens (133). In China, the HPAIV backyard chicken–wild bird interface was a densely populated, large-scale wetland system, which incorporated farmed waterfowl, wild waterfowl, and free-ranging backyard chickens (134). In Thailand, backyard chicken farms consist of free-ranging native chickens and fighting cocks, which interact with wild birds in nearby ponds or canals (135). In Mali, the NDV–AIV backyard chicken–wild bird interface was located within the Inner Niger Delta, where backyard chickens free ranged during the day but resided in rural village households at night (136). In Argentina, the LPAIV backyard–chicken interface comprised 22 species of waterfowl and shorebirds that shared a wetland habitat with backyard chickens (137).

Thus, when characterizing the backyard chicken–wild bird interface, one of the most important, yet understated, components is habitat. It not only influences the composition of the avian community but also the presence of wild bird species that are susceptible to a particular pathogen. Therefore, in defining the backyard chicken wild bird interface, we define it as a habitat in which susceptible or infectious wild birds overlap in land use with susceptible or infectious backyard chickens. In the literature review below, we discuss instances of spillover or suspected spillover of “backyard chicken” pathogens into susceptible wild birds.

### Literature Review

We searched for studies describing potential pathogen spillover from backyard chickens to wild birds. We also conducted searches for studies on the occurrence of pathogens common to backyard chickens that are also found in wild birds, where spillover has not yet been documented, but the potential for such spillover exists. Both searches utilized the Google Scholar and Web of Science databases. In our search strategy, we included pathogens in which chickens serve as the primary or reservoir host to demonstrate evidence of spillover. We used the following search terms and Boolean operators: “pathogen of interest” OR “pathogen and disease” OR “pathogen infect^*^” and “wild bird” OR “wild birds” OR “spillover” OR “feral birds” (*n* = 28,110). We excluded experimental inoculation studies, research that had not undergone peer review, studies concerning commercial poultry, and we also excluded any study in which the primary host and the recipient host could not be determined (exclusion criteria *n* = 27,878). For example, many bacterial species are shared between wild birds and backyard chickens (e.g., *Salmonella* spp.). Thus, we only included that pathogen/study in the review portion of the paper when the pathogens were host specific or when laboratory analyses, such as sequencing, could identify the most likely primary host. In Supplemental Table 2, we provide a list of pathogens shared between both groups, but where insufficient data for spillover events were available. Finally, while our review is generally USA-centric, we drew examples from the global literature to provide a comprehensive assessment of pathogen spillover from backyard chickens into wild birds.

### Pathogen Transmission to Wild Birds

Pathogen transmission dynamics at the poultry–free-living bird interface are not only subject to within-host ecological and evolutionary pressures (138) but also, most importantly, are frequently bidirectional in nature (27, 139, 140). The establishment of “open-air” or “free-range” poultry habitats that overlap with wild bird habitats is a key step in the loss of sympatric species contact barriers, which may facilitate the transmission of opportunistic microbes (132, 141–143). The introduction of high densities of poultry into a diverse, susceptible avian community is likely to accelerate wild and domestic bird encounter rates with generalist pathogens. For example, the most commonly cited group of viruses that follow this model are the HPAI subtypes (144, 145). Repeated transmission events of generalist, multihost pathogens into a recipient species, in this case, domestic chickens, from wild bird spillover hosts may lead into pathogen establishment and spillback from backyard poultry (146). This has the potential to establish a positive feedback cycle among susceptible wild birds and domestic chickens; such has occurred over the last two decades with the H5N1 goose/Guangdong (GsGD) lineage of HPAI (147–150).

It is well-established that backyard chickens may serve as pathogen reservoirs to the commercial poultry industry (151, 152) and that the most likely mechanism of spillover involves wild birds (139). For example, Lebarbenchon et al. (153) hypothesized that small passerines served as bridge hosts for the H7N9 virus low-pathogenic avian influenza virus (LPAI) from infectious waterfowl to US commercial turkey houses. However, some of those wild bird species may also be species of special concern as defined the by the IUCN (154).

Although much of the literature has examined the risks that wild birds pose to backyard chickens and commercial poultry (74, 132, 155–157), a few studies have examined the converse. Backyard chickens may not only serve as a biosecurity risk to commercial flocks but also as a reservoir for the spillover of common “poultry” pathogens to wild birds (120, 158). For example, on the Galápagos Island of Santa Cruz, Soos et al. (121) found that backyard poultry had a high prevalence of seropositive chickens for six common “poultry” pathogens, although all nearby wild birds that were tested were seronegative (121).

However, a similar study was conducted on the Galápagos island of Floreana, which has a much longer history of anthropogenic and agricultural modification than Santa Cruz. In this case, surveys of endemic wild bird found serological evidence against NDV, avian poxvirus, and avian adenovirus-2 (119). As a result of the Floreana study, the risk of pathogen transmission from backyard chickens was considered too high to attempt reintroduction efforts of endangered, endemic Galápagos wild bird species (119). These observations are significant for the endangered species repatriation across all wildlife refuges, as pathogen transmission from backyard chickens has the potential to extend beyond the borders of protected habitats (159, 160).

Perhaps the best example in which the transmission of a bacterial pathogen from chickens to wild birds has been documented is *Mycoplasma gallisepticum* (MG). MG spilled over from poultry in 1994 into house finches (*Haemorhous mexicanus*) and rapidly became endemic in North American passerine species (161, 162). A related species, *Mycoplasma synoviae*, which is also commonly detected in backyard chickens (163, 164), has also occasionally been isolated from passerines and near-passerine species (165, 166). In one instance, an isolate with genetic similarities to an *M. synoviae* vaccine strain was isolated from a captive lesser flamingo (*Phoeniconaias minor*) in Northern Italy (167).

Other notable examples of bacterial pathogens known to originate from chickens and/or backyard turkeys include the Gram-negative bacterium *Bordetella avium*, which has been isolated from waterfowl, psittacines, and passerines in the eastern United States of America (168). Although *Bordetella avium* is most commonly associated with bordetellosis in commercial turkeys (169, 170), it is also frequently found in backyard and wild turkeys (171, 172). *Pasteurella multocida* is a pathogen known for its acute nature and substantial mortality in both chickens and waterbirds (173, 174) and has a seemingly global distribution in backyard chickens, having been reported from areas such as Upper Egypt (175), Ethiopia (176), Zimbabwe (112), Tanzania (177), Denmark (178), California (113), and India (179). The spillover and potential spillback of *P. multocida* ssp. *multocida* between backyard flocks and wild waterfowl, cormorants, and shorebirds were documented in the late 1990's in Denmark (180).

Viral pathogens, due to their pathogenicity, have been better studied. Newcastle disease (ND), caused by virulent Newcastle disease virus (NDV) strains is among one of the most significant pathogens at the backyard poultry–wild bird interface (49). It is a highly contagious, acute, and systemic illness primarily known to afflict poultry; however, clinical symptoms have also been documented among bird species outside of the Galliformes family (181, 182). NDV has been isolated from a broad range of avian hosts, and it is now generally presumed that all bird species are susceptible to the replication, shedding, and transmission of the virus (183–186). NDV is also often endemic among the backyard flocks of developing nations (187). For example, in Vietnam, up to 34% of unvaccinated backyard chickens tested positive for antibodies against NDV (188). In Bushehr province, Iran, 40% of unvaccinated chickens tested positive for antibodies against NDV (189). In a review of NDV vaccine spillover events, Ayala et al. found that 9.3% of spillover events involved a wild bird belonging to a species listed by the IUCN as either in decline or in an eminent threat of decline (154, 190). In Latin America, free-ranging backyard flocks have been investigated as potential sources of pathogen spillover into resident and endemic wild birds, including NDV (120, 129, 130). Across four African countries, Cappelle et al. (191) found that 8.9% of the species that tested positive for NDV by real-time PCR were listed by the IUCN as either vulnerable or near threatened.

Avian encephalomyelitis virus is a picornavirus with a worldwide distribution that infects juvenile chickens, pheasants, quail sand turkeys, including juvenile backyard chickens (112, 192). It has also been reported in songbirds of the Paridae family (192), wild turkeys (*Meleagris gallopavo*) in the Southeastern United States (193), and rock pigeons (*Columba livia*) in Turkey (194). Corvids appear to be particularly susceptible to infection with avian reovirus (195), a poultry pathogen also commonly detected in backyard chickens (112, 196). In addition, two die-offs of American Woodcocks (*Scolopax minor*) were attributed to the virus (197).

Avian lymphoid leukosis virus has been isolated from backyard chickens (113), as well as both captive and wild bird species, including passerines, columbids, waterfowl, and psittacines (198–202). Fowl adenovirus-4 (FAdV-4), an emerging pathogen of poultry, was isolated from rock pigeons of Hong Kong and black kites (*Milvus migrans*) of Kashipur, India (203–205). IBV, a gamma-coronavirus of poultry and backyard chickens (206), has been isolated from waterfowl and wading birds of Beringia, Alaska, and the nation of Poland (207). IBDV is an immunosuppressive virus of poultry and backyard chickens that targets B-lymphocytes and associated tissues of the immune system (208–210). It has been isolated from a wide variety of bird species, including waterfowl, columbids, passerines, Galliformes, and members of the Charadriiformes (211–214).

Marek's disease virus is a neoplastic virus in the Herpesviridae family, which is ubiquitous among backyard chicken flocks and poultry worldwide (215). The virus or antibodies against the virus have been detected in captive members of the Galliformes and wild waterfowl, including endangered lesser white-fronted Geese (*Anser erythropus*) (216–219). Reticuloendotheliosis viruses (REV) are a group of retroviruses and the causative agent of reticuloendotheliosis, an immunosuppressive and neoplastic disorder of poultry and backyard chickens (112, 220). The virus has been isolated from Galliformes, waterfowl, columbids, and endangered Attwater's prairie chickens (*Tympanuchus cupido attwateri*) (221–223).

MacQueen's bustards (*Chlamydotis macqueenii*) are categorized as vulnerable by the IUCN (224). Illegally trapped and transported individuals were found to have been exposed to NDV, avian poxvirus, and *Chlamydia* spp.; pathogens were also found in backyard chickens (188, 225–227). In Burkina Faso, three hooded vultures (*Necrosyrtes monachus*), a western plantain-eater (*Crinifer piscator*), and an Ovambo sparrowhawk (*Accipiter ovampensis*) were infected with various combinations of HPAI, NDV, and IBV (228). Given that hooded vultures are categorized as critically endangered by the IUCN, this finding is extremely significant (229). Similarly, Eurasian oystercatchers (*Haematopus ostralegus*) are listed by the IUCN as vulnerable in Europe (230) and in the United Kingdom; 12% of sampled Eurasian oystercatchers, along with various waterfowl and shorebirds were found to be infected with IBV (231). Several species of Antarctic penguins, including the near-threatened emperor penguin (*Aptenodytes forsteri*), have been found to be infected with *Chlamydia* spp., LPAI, NDV, and IBDV (232). A die-off of reintroduced, endangered whooping cranes (*Grus americana*) in Florida was attributed to IBDV, serotype 2 (233). Last, a low-virulent NDV strain was isolated from two bald eagles (*Haliaeetus leucocephalus*) and one great horned owl (*Bubo virginianus*) in Minnesota during the winter of 2009 (234).

Even given all these reports, pathogen transmission between backyard poultry and native birds remain only causally linked and likely underreported (190). For example, the inclusion criteria for this review and Supplemental Table 2 yielded 232 papers. However, after accounting for duplicate reports, only 11 papers remained. These remaining studies provided evidence of pathogen spillover to and from domestic chickens and wild birds using genomic comparisons and molecular epidemiology techniques. Economically significant viruses such as HPAIV and NDV were highly represented (50, 139, 141, 146, 158, 190, 235–237), whereas bacterial and parasitic species were less so (141, 162, 180). A contributing factor to this scarcity of evidence is likely that the mechanisms of transmission differ across pathogens and avian host species (238, 239). Consequentially, conclusive evidence for pathogen host shifts has generally only been reconstructed with molecular techniques after enzootics have resulted in high mortality rates [i.e., *Mycoplasma gallisepticum* in Fringillids (162)]. Moreover, the same molecular techniques have also clarified the origins of pathogens once attributed to chickens. For example, canarypox has been identified as the causative agent of disease in Hawaiian and Galápagos avifauna as opposed to fowlpox viruses that originated from chickens, as previously believed (240, 241).

It has become clear that backyard poultry play a role in the transmission of potentially virulent pathogens (56, 242, 243), yet their impact on wild bird populations remains largely unknown. Specifically, it is the absence of cross-species barriers between backyard chickens and native birds that may exacerbate pathogen transmission. When gregarious and social native bird species overlap in habitat with high-density chicken operations, the ecological barriers to pathogen transmission are lost (244). This scenario pertains to peridomestic birds that consume free-ranging backyard chicken feed and water sources, which may then interact with other wild bird species that would have otherwise remained unexposed (Ayala et al. unpublished data 2020) (245–247).

### Backyard Chickens and Biosecurity

Backyard flocks are implicated in maintaining enzootics of two critically important RNA viruses around the globe, NDV and HPAIV (237, 248–255). This is largely due to essential differences in the biosecurity of backyard flocks and commercial flocks (238, 256). For instance, while the commercial industry practices high containment and mass immunization against NDV, only 3% to 10% of backyard flocks are immunized for common poultry pathogens (103, 257, 258). Backyard flocks are also often subject to little to no biosecurity regulations, where biosecurity protocols and vaccination serve as the essential management practices that mitigate the transfer of infectious agents into and from the flock (238).

For backyard chickens, biosecurity and vaccination lead to healthier and more productive backyard chicken operations—and these benefits apply across global poultry operations. For example, in Mozambique, backyard flocks that were vaccinated against NDV with the thermostable I-2 vaccine had a higher hatch rate during brooding than those who were not vaccinated (259). In Ethiopia, up to 50% of backyard chicken flocks suffer mortality from infection by NDV (260). A biosecurity risk assessment found that large flock sizes, reduced cleaning frequencies, and water sources that were shared with other flocks were significant predictors for increased NDV incidence (261). In Thailand, the greatest risk factor associated with increased HPAI H5N1 flock incidence included the trade of live chickens between backyard flocks, while the use of disinfectants during a cleaning regimen reduced transmission (135). In Maryland, AIV seropositivity in backyard flocks was associated with proximity to waterfowl, while the use of pest control was associated with a reduced likelihood of seropositivity (131). In Bangladesh, increased HPAI H5N1 incidence in backyard flocks was associated with feeding backyard chickens offal from slaughtered birds, allowing contact with rock pigeons, and living near a body of water. However, the likelihood of HPAI H5N1 incidence was reduced when chickens were separated from waterfowl (262). In Oman, between 84 and 90% of backyard flocks were seropositive for AIV and NDV, respectively. Flock contact with wild birds, the presence of water bodies, high human densities, and the presence of live bird markets were proposed as explanatory variables for AIV and NDV (263).

Thus, in addition to a lack of regulation, in some areas, vaccination, hygienic measures, and biosecurity compliance remain logistically prohibitive, which means that backyard chickens and live bird markets may remain sources of transmissible pathogens (264, 265). Moreover, limitations in husbandry practices are sometimes the result of finite resources. In these cases, clinical illness may be overlooked due to limited experience with disease, or otherwise unreported, for fear of mandatory flock culling (81, 104). Even more worrisome is the practice of covertly transporting and selling sick fowl or discarding infected eggs and carcasses into the environment. Such practices have been attributed to maintaining cyclic NDV among unvaccinated flocks (266).

Surveys of backyard chicken owners suggest that extension education should focus on management practices associated with disease transmission, such as carcass disposal, coop cleaning regimens, and the proximity of wild birds (97, 116, 267). Even in developed countries, with ample access to education and resources, backyard chickens remain a concern. For example, in the United States, ~30% of backyard poultry owners maintain wild bird feeders, increasing contact rates to free-living birds within infectious environments (258). There, backyard flock owners span socioeconomic, geographical, and community types [i.e., urban, rural, and suburban (267)], and the average flock size per household varies from 25 to 49 birds (96, 268). With the exception of local zoning regulations, backyard chicken laws remain primarily unregulated and/or unenforced. In addition, poultry specialists, extension veterinarians, and community educators increasingly lack the resources needed to monitor the continuously expanding and vulnerable backyard poultry sector (268).

In the United States, backyard chickens are increasing in popularity, and between 2 and 7.4% of US without chickens plan to own them within 5 years (96). As the backyard chicken industry continues to expand, the issues are pressing further to the surface. Practices such as inconsistent husbandry management (103), poor vaccination compliance (258), and a reduction in national poultry “herd immunity (269)” perpetuate disease vulnerability to the commercial food supply and to native bird communities. Wet markets, the illicit pet bird trade, and poultry trafficking have instigated low-pathogenic avian influenza outbreaks in the Northeastern United States (270, 271) and were responsible for the 1972 (272), 2002 (273), and current virulent NDV outbreaks in California (274). In 2013, the Food and Agriculture Organization of the United Nations identified “smallholder livestock systems” as disproportionately large facilitators of infectious disease spread (275).

Critical events, such as the 2014–2015 HPAIV H5Nx outbreak in the United States not only leads to the loss of public confidence in food security but also further decimates the agricultural economy through mass depopulation, quarantines, and trade embargos (276, 277). In addition, while LPAI is commonly transmitted by wild birds, not all wild bird species are adapted to HPAI, and morbidity or mortality may result in some wild individuals (277–279). For example, in the 2014–2015 H5Nx outbreak, some North American waterfowl, shorebirds, and even raptors died from the pathogen (256).

In the United States, outbreaks of low-pathogenic avian influenza (LPAI) H7 variants in live bird markets were sporadically detected in the mid-Atlantic and Northeastern areas between 1986 and 2004. These occurred primarily in New York, New Jersey, Massachusetts, Rhode Island, Connecticut, and Pennsylvania (270, 271, 280). Upon investigating an extensive outbreak of HPAI in Houston, Texas, in 2004, the evidence suggests that it also originated from a live bird market (281). In eastern Texas, an earlier survey of backyard poultry flocks found that 100% of each flock harbored individuals with antibodies to IBDV (282). Such events demonstrate that backyard chickens and the movement of live poultry not only pose a risk to native birds but to the commercial poultry industry as well (116).

## Conclusions and Recommendations

Although spillover and spillback at the poultry–avifaunal interface has been documented, for many pathogens, the underlying reasons that facilitate spillover and spillback remain confounded. Recommendations are provided here, but they are not exhaustive, and should be tailored to the local circumstances, flock types, laws, and regulations (283). Arzey et al. (283) provide a thorough review of backyard poultry recommendations for small-scale producers, which are easily adapted to the backyard flock owner.

Pathogen spillover may be a result of poor bioexclusion, such as the failure to quarantine new flock individuals or inadequate coop enclosures that do not separate wild birds from backyard chickens (273, 284). For instance, biosecurity and quarantine protocols are two of the most common measures undertaken by the poultry industry to reduce pathogen transmission from backyard chickens and wildlife (285). Restricting the movements of infectious birds or equipment, including transport, is imperative to reduce transmission to susceptible individuals—into both chickens and wild bird species (283, 286). Poor vaccine compliance is also a failure of disease management, specifically because vaccination can reduce clinical signs, and the amount of virus shed from an infected chicken that was previously vaccinated (287–289).

Backyard flock owners should not maintain wild bird feeders on their properties, thereby inhibiting visits from large flocks of multiple wild avian species, which may include susceptible or even infectious wild birds (103). Backyard chicken feeders should be kept where only chickens can reach them, while mesh should be utilized wherever possible to prevent wild birds from interacting with chickens, their coops, or their resources (290). Removing contaminated water sources, insects, and rodents reduces point sources of pathogen contamination, not just to other chicken flocks but also to wild birds (283). Increased attention to owner and visitor hygiene [i.e., the changing of footwear when visiting different flocks, and limiting the number of visitors to backyard coops], are important principles of bioexclusion and biocontainment (103, 257). Last, dead birds or eggs that are suspected of contamination should be disposed of in a manner that complies with local guidelines, for example, incineration or, if applicable, burial, according to environmental guidelines (283). In the case of an unexplained death and especially if infectious diseases are suspected in a mortality event, submission of carcasses and samples to approved diagnostic labs with experience in avian cases for testing is highly recommended (291).

Backyard flock owners increasingly report emotional ties to their chickens (258, 292). Thus, the inclusion of standard avian veterinary care and the management of common chicken pathogens into flock routines are realistic objectives in nations like the United States (108, 111). However, in the rural areas of developing nations, where backyard chickens are kept strictly as livestock, these recommendations may appear impractical for the small-scale flock owner (238). Backyard chickens in these areas, also known as village chickens, are low-input, low-output agricultural models that serve as more than a source of protein (90). Meat and eggs from these chickens can be bartered or sold, providing much needed income for other needs such as medicine, clothing, and school fees (293). Biosecurity implementation may increase the required input of the system, affecting profits, reducing food security, and lessening overall benefits for small-scale flock owners (238, 294). However, pathogen management and biosecurity in such areas is crucial; for example, in India, backyard chickens are often utilized to ensure a steady food supply in the case of crop failures (295). For these reasons, low-cost biosecurity measures should be implemented at the community level (296). This not only ensures a steady source of protein for residents but also may mitigate the potential for a spillover–spillback pathogen transmission feedback loop into and from wild birds (129).

Low-cost biosecurity recommendations for backyard chickens in rural communities includes breeding individuals that are resistant to locally prevalent pathogens, biosecurity educational programs, improving local hygiene, the culling of sick individuals, and the use of thermostable, low-cost vaccines (294–296). For instance, flock rotation is commonly utilized in Australia to reduce the prevalence of endoparasites, such as coccidia (281). In addition, effectively implementing biosecurity beyond the community level likely includes a regional assessment of critical backyard chicken pathogens, their mechanisms of spread, and a methodology to interrupt those pathways (38). Regional biosecurity planning should likely involve educational, financial, and regulatory interventions at a governmental level.

As previously discussed, pathogen spillover is ultimately driven through the ecological loss of natural barriers between species. When multiple species of the same phylogenetic order are thrust into sharing the same habitat, spillover between those species is likely to occur. Moreover, certain behaviors in domestic and wild birds lend themselves to increasing that probability, such as being foraging generalists, ground foragers, or flocking species. However, in the literature on the poultry–avifaunal interface, there remains a paucity of experimentally and empirically derived field data regarding the bidirectional potential of poultry–avifaunal pathogens outside of HPAI.

Bidirectional potential includes the ability of the *collective* wild and domestic avian host community to maintain a pathogen traditionally associated with poultry above the threshold levels required to continue an outbreak. Some pathogens have been extensively studied in the field, laboratory, and through mathematical models (i.e., avian influenza). This is likely due to the zoonotic potential and public health risk of the virus, in addition to the economic burden following a commercial or village poultry outbreak (297–300). However, even current surveillance methods for HPAI among reservoir avian species have been reported as “unsatisfactory” when contrasted against the efforts applied toward understanding human-to-human transmission (301). Further research is needed into the backyard chicken–wild bird interface, especially near sites such as Important Bird Areas (IBPs), where the conservation of declining species is a priority.

## Author Contributions

AA, MY, and SH: conceptualization, methodology, project administration, resources, and writing—review and editing. AA and SH: funding acquisition, visualization, and writing—original draft. MY and SH: supervision. All authors contributed to the article and approved the submitted version.

## Conflict of Interest

The authors declare that the research was conducted in the absence of any commercial or financial relationships that could be construed as a potential conflict of interest.
