# Supplementary material for: A Review of Pathogen Transmission at the Backyard Chicken–Wild Bird Interface
Source: Front Vet Sci. 2020 Sep 24;7:539925. doi: 10.3389/fvets.2020.539925 (PMC7541960; doi:10.3389/fvets.2020.539925)
Supplement: Supplementary file 1 [file Table_1.DOCX]

Supplemental Table 1: Sax et al 2006 identified 18 species of primarily endemic island-species that were declared extinct, or extinct in the wild due to infectious diseases coupled with invasive species displacement, spanning from 1837 to 1989. An additional 77 wild bird species were declared currently at risk by the International Union for the Conservation of Nature (IUCN) and Birdlife International due to infectious diseases and are incorporated in this table.

| **Specific epithet** | **Common Name** | **Family** | **Pathogen** | **Geographic Range** | **Assessment Year** | **IUCN Status** | **Citation** |
| --- | --- | --- | --- | --- | --- | --- | --- |
| *Acrocephalus brevipennis* | Cape Verde warbler | Acrocephalidae | Unspecified | Cape Verde Islands | 2017 | VU | (IUCN 2020) |
| *Agapornis nigrigenis* | Black-cheeked Lovebird | Psittaculidae | Beak and feather disease | Zambia | 2016 | VU | (IUCN 2020) |
| *Akialoa ellisiana* | Oʻahu ʻakialoa | Fringillidae | Unspecified | Hawaiian Islands, USA | 1837, 2017 | EX | (Smith, Sax et al. 2006, IUCN 2020) |
| *Akialoa lanaiensi* | Lana'i 'akialoa | Fringillidae | Unspecified | Hawaiian Islands, USA | 1892, 2017 | EX | (Smith, Sax et al. 2006, IUCN 2020) |
| *Akialoa obscura* | Lesser ʻakialoa | Fringillidae | Unspecified | Hawaiian Islands, USA | 1940, 2016 | EX | (Smith, Sax et al. 2006) |
| *Akialoa stejnegeri* | Kauaʻi ʻakialoa | Fringillidae | Unspecified | Hawaiian Islands, USA | 1969, 2017 | EX | (Smith, Sax et al. 2006, IUCN 2020) |
| *Alopecoenas sanctaecrucis* | Santa Cruz Ground Dove | Columbidae | Trichomoniasis | Solomon Islands, Vanuatu | 2018 | EN | (IUCN 2020) |
| *Aphelocoma coerulescens* | Florida Scrub-jay | Corvidae | West Nile virus | Florida, USA | 2017 | VU | (IUCN 2020) |
| *Ardeotis nigriceps* | Great Indian Bustard | Otididae | Unspecified | India | 2018 | CR | (IUCN 2020) |
| *Ara glaucogularis* | Blue-throated Macaw | Psittacidae | *Philornis* spp. | Bolivia | 2018 | CR | (IUCN 2020) |
| *Atelornis crossleyi* | Rufous-headed Ground Roller | Brachypteraciidae | *Haemoproteus forresteri*  *Leucocytozoon frascai* | Madagascar | 2016 | NT | (Savage and Greiner 2004, IUCN 2020) |

| **Specific epithet** | **Common Name** | **Family** | **Pathogen** | **Geographic Range** | **Assessment Year** | **IUCN Status** | **Citation** |
| --- | --- | --- | --- | --- | --- | --- | --- |
| *Bucorvus leadbeateri* | Southern Ground-hornbill | Bucorvidae | Newcastle disease virus | Southern Africa | 2016 | VU | (IUCN 2020) |
| *Calidris canutus* | Red Knot | Scolopacidae | Avian Influenza virus (H5N1 subtype) | North and South America | 2018 | NT | (IUCN 2020) |
| *Centrocercus minimus* | Gunnison Sage- Grouse | Phasianidae | West Nile virus | Western United States | 2016 | EN | (IUCN 2020) |
| *Centrocercus urophasianus* | Greater Sage- Grouse | Phasianidae | West Nile virus | Midwestern North America | 2016 | NT | (IUCN 2020) |
| *Chasiempis ibidis* | O'ahu 'Elepaio | Monarchidae | *Plasmodium relictum*  Avipoxvirus | Hawaiian Islands (USA) | 2016 | EN | (IUCN 2020) |
| *Chasiempis sclateri* | Kauaʻi ʻElepaio | Monarchidae | *Plasmodium relictum*  Avipoxvirus | Hawaiian Islands (USA) | 2018 | VU | (IUCN 2020) |
| *Chasiempis sandwichensis* | Hawai`i ʻElepaio | Monarchidae | *Plasmodium relictum*  Avipoxvirus  *Phthiraptera* spp. | Hawaiian Islands (USA) | 2016 | VU | (IUCN 2020) |
| *Chlorodrepanis stejnegeri* | Kauaʻi ʻAmakihi | Fringillidae | *Plasmodium relictum*  Avipoxvirus | Hawaiian Islands (USA) | 2016 | VU | (IUCN 2020) |
| *Clangula hyemalis* | Long-tailed Duck | Anatidae | Avian Cholera  Avian Influenza Virus (H5N1 subtype) | Northern Hemisphere | 2018 | VU | (IUCN 2020) |
| *Coracopsis barklyi* | Seychelles Parrot | Psittrichasidae | Beak and Feather disease | Seychelles Islands | 2016 | VU | (IUCN 2020) |
| *Corvus hawaiiensis* | Hawaiian Crow | Corvidae | West Nile virus  *Plasmodium relictum*  *Toxoplasma gondii*  Avipoxvirus | Hawaiian Islands (USA) | 2016 | EW | (Smith, Sax et al. 2006, IUCN 2020) |
| *Coturnix novaezelandiae* | New Zealand Quail | Phasianidae | Unspecified | New Zealand | 1875, 2016 | EX | (Smith, Sax et al. 2006, IUCN 2020) |
| *Cyanoramphus malherbi* | Malherbe's Parakeet | Psittaculidae | Beak and Feather Disease | New Zealand | 2018 | CR | (IUCN 2020) |

| **Specific epithet** | **Common Name** | **Family** | **Pathogen** | **Geographic Range** | **Assessment Year** | **IUCN Status** | **Citation** |
| --- | --- | --- | --- | --- | --- | --- | --- |
| *Drepanus pacifica* | Hawaii Mamo | Drepanididae | Unspecified | Hawaiian Islands (USA) | 1899, 2016 | EX | (Smith, Sax et al. 2006, IUCN 2020) |
| *Ectopistes migratorius* | Passenger Pigeon | Columbidae | Newcastle disease virus | Eastern North America | 1914, 2019 | EX | (Smith, Sax et al. 2006, IUCN 2020) |
| *Eudyptes chrysocome* | Southern Rockhopper Penguin | Spheniscidae | Avian cholera  Unspecified | Southern Hemisphere | 2018 | VU | (IUCN 2020) |
| *Eudyptes chrysolophus* | Macaroni Penguin | Spheniscidae | Avian Cholera | Southern Hemisphere | 2018 | VU | (IUCN 2020) |
| *Eudyptes moseleyi* | Northern Rockhopper Penguin | Spheniscidae | Avian Cholera | Southern Hemisphere | 2018 | EN | (IUCN 2020) |
| *Eudyptes pachyrhynchus* | Fiordland Penguin | Spheniscidae | *Leucocytozoon* spp. | New Zealand | 2018 | VU | (IUCN 2020) |
| *Eudyptes schlegeli* | Royal Penguin | Spheniscidae | Unspecified | Macquarie Island (Australia) | 2018 | NT | (IUCN 2020) |
| *Eunymphicus cornutus* | Horned Parakeet | Psittaculidae | Beak and feather disease | New Caledonia | 2016 | VU | (IUCN 2020) |
| *Fregilupus varius* | Reunion Starling | Sturnidae | Unspecified | Reunion Island | 1850, 2016 | EX | (Smith, Sax et al. 2006, IUCN 2020) |
| *Geospiza heliobates* | Mangrove Finch | Thraupidae | *Philornis downsi*  Avipoxvirus | Galápagos islands (Ecuador) | 2018 | CR | (IUCN 2020) |
| *Geospiza pallida* | Woodpecker Finch | Thraupidae | *Philornis downsi*  Avipoxvirus | Galápagos islands (Ecuador) | 2016 | VU | (IUCN 2020) |
| *Geospiza pauper* | Medium Tree-finch | Thraupidae | *Philornis downsi*  Avipoxvirus | Galápagos islands (Ecuador) | 2018 | CR | (IUCN 2020) |
| *Geospiza psittacula* | Large Tree-finch | Thraupidae | *Philornis downsi*  Avipoxvirus | Galápagos islands (Ecuador) | 2016 | VU | (IUCN 2020) |
| *Gymnogyps californianus* | California Condor | Cathartidae | West Nile virus | Western United States | 2018 | CE | (IUCN 2020) |

| **Specific epithet** | **Common Name** | **Family** | **Pathogen** | **Geographic Range** | **Assessment Year** | **IUCN Status** | **Citation** |
| --- | --- | --- | --- | --- | --- | --- | --- |
| *Hemignathus affinis* | Maui Nukupuʻu | Fringillidae | *Plasmodium relictum*  Avipoxvirus | Hawaiian Islands (USA) | 2016 | CR | (IUCN 2020) |
| *Hemignathus hanapepe* | Kauai Nukupuʻu | Fringillidae | *Plasmodium relictum*  Avipoxvirus | Hawaiian Islands (USA) | 2018 | CR | (IUCN 2020) |
| *Hemignathus lucidus* | Oʻahu Nukupuʻu | Fringillidae | *Plasmodium relictum*  Avipoxvirus | Hawaiian Islands (USA) | 1899, 2016 | EX | (IUCN 2020) |
| *Hemignathus obscurus* | Lesser ʻakialoa | Fringillidae | Unspecified | Hawaiian Islands (USA) | 1940, 2017 | EX | (Smith, Sax et al. 2006, IUCN 2020) |
| *Hemignathus wilsoni* | Akiapolaau | Fringillidae | *Plasmodium relictum*  Avipoxvirus | Hawaiian Islands (USA) | 2016 | EN | (IUCN 2020) |
| *Hesperiphona vespertina* | Evening Grosbeak | Fringillidae | Unspecified | North America | 2018 | VU | (IUCN 2020) |
| *Heteralocha acutirostris* | Huia | Callaeidae | Unspecified | New Zealand | 1907, 2017 | EX | (Smith, Sax et al. 2006, IUCN 2020) |
| *Lanius ludovicianus* | Loggerhead Shrike | Laniidae | West Nile virus | North America | 2017 | NT | (IUCN 2020) |
| *Lathamus discolor* | Swift Parrot | Psittacidae | Beak and Feather Disease | Australia | 2018 | CR | (IUCN 2020) |
| *Limosa lapponica* | Bar-tailed Godwit | Scolopacidae | Avian Influenza virus (H5N1 subtype) | Global | 2017 | NT | (IUCN 2020) |
| *Loxioides bailleui* | Palila | Fringillidae | *Plasmodium relictum*  Avipoxvirus | Hawaiian Islands (USA) | 2016 | CR | (IUCN 2020) |
| *Loxops caeruleirostris* | ʻAkekeʻe | Fringillidae | *Plasmodium relictum*  Avipoxvirus | Hawaiian Islands (USA) | 2018 | CR | (IUCN 2020) |
| *Loxops coccineus* | Hawaii Akepa | Fringillidae | *Plasmodium relictum*  Avipoxvirus | Hawaiian Islands (USA) | 2016 | EN | (IUCN 2020) |
| *Loxops ochraceus* | Maui Akepa | Fringillidae | *Plasmodium relictum*  Avipoxvirus | Hawaiian Islands (USA) | 2018 | CR | (IUCN 2020) |
| *Magumma parva* | Anianiau | Fringillidae | *Plasmodium relictum*  Avipoxvirus | Hawaiian Islands (USA) | 2016 | VU | (IUCN 2020) |
| *Manucerthia mana* | Hawaiʻi creeper | Fringillidae | *Plasmodium relictum*  Avipoxvirus | Hawaiian Islands (USA) | 2016 | EN | (IUCN 2020) |

| **Specific epithet** | **Common Name** | **Family** | **Pathogen** | **Geographic Range** | **Assessment Year** | **IUCN Status** | **Citation** |
| --- | --- | --- | --- | --- | --- | --- | --- |
| *Megadyptes antipodes* | Yellow-eyed Penguin | Spheniscidae | *Leucocytozoon* spp.  *Corynebacterium* spp | New Zealand | 2018 | EN | (IUCN 2020) |
| *Melamprosops phaeosoma* | Po’o-uli | Fringillidae | *Plasmodium relictum*  Avipoxvirus | Hawaiian Islands (USA) | 2006, 2019 | EX | (IUCN 2020) |
| *Mimus melanotis* | San Cristobal Mockingbird | Mimidae | *Plasmodium relictum*  Avipoxvirus  *Philornis downi*  West Nile virus | Galápagos islands (Ecuador) | 2016 | EN | (IUCN 2020) |
| *Mimus trifasciatus* | Floreana Mockingbird | Mimidae | Avipoxvirus | Galápagos islands (Ecuador) | 2018 | EN | (IUCN 2020) |
| *Moho apicalis* | O'ahu 'ō'ō | Mohoidae | Unspecified | Hawaiian Islands (USA) | 1850, 2017 | EX | (Smith, Sax et al. 2006, IUCN 2020) |
| *Moho bishopi* | Bishop's 'ō'ō | Mohoidae | Unspecified | Hawaiian Islands (USA) | 1981, 2016 | EX | (Smith, Sax et al. 2006, IUCN 2020) |
| *Moho braccatus* | Kauaʻi ʻōʻō | Mohoidae | Unspecified | Hawaiian Islands (USA) | 1987, 2016 | EX | (Smith, Sax et al. 2006, IUCN 2020) |
| *Moho nobilis* | Hawaiʻi ʻōʻō | Mohoidae | Unspecified | Hawaiian Islands (USA) | 1934, 2016 | EX | (Smith, Sax et al. 2006, IUCN 2020) |
| *Myadestes myadestinus* | Kāmaʻo | Turdidae | Unspecified | Hawaiian Islands (USA) | 1989, 2016 | EX | (Smith, Sax et al. 2006, IUCN 2020) |
| *Myadestes palmeri* | Puaiohi | Turdidae | *Plasmodium relictum*  Avipoxvirus | Hawaiian Islands (USA) | 2018 | CR | (IUCN 2020) |
| *Nannopterum harrisi* | Flightless Cormorant | Phalacrocoracidae | *Toxoplasma gondii* | Galápagos islands (Ecuador) | 2018 | VU | (IUCN 2020) |
| *Necrosyrtes monachus* | Hooded Vulture | Accipitridae | Avian Influenza virus (H5N1 subtype) | Sub Saharan Africa | 2017 | CE | (IUCN 2020) |
| *Neophema chrysogaster* | Orange-bellied Parrot | Psittaculidae | Beak and Feather Disease | Southern Australia | 2018 | CE | (IUCN 2020) |
| *Neophron percnopterus* | Egyptian Vulture | Accipitridae | Avipoxvirus | Europe, Southeast Asia, Africa | 2019 | EN | (IUCN 2020) |

| **Specific epithet** | **Common Name** | **Family** | **Pathogen** | **Geographic Range** | **Assessment Year** | **IUCN Status** | **Citation** | |  |
| --- | --- | --- | --- | --- | --- | --- | --- | --- | --- |
| *Nesoenas mayeri* | Pink Pigeon | Columbidae | *Trichomonas gallinae* *Leucocytozoon marchouxi* | Mauritius | 2018 | VU | | (IUCN 2020) | |
| *Numenius arquata* | Eurasian Curlew | Scolopacidae | Avian Influenza virus (H5N1 subtype) | Europe, Asia, Africa | 2017 | NT | | (IUCN 2020) | |
| *Oreomystis bairdi* | ‘Akikiki | Fringillidae | *Plasmodium relictum*  Avipoxvirus | Hawaiian Islands (USA) | 2018 | CR | | (IUCN 2020) | |
| *Paroreomyza flammea* | Kākāwahie | Fringillidae | Unspecified | Hawaiian Islands (USA) | 1963, 2017 | EX | | (Smith, Sax et al. 2006, IUCN 2020) | |
| *Paroreomyza maculate* | O'ahu 'Alauahio | Fringillidae | *Plasmodium relictum*  Avipoxvirus | Hawaiian Islands (USA) | 2016 | CR | | (IUCN 2020) | |
| *Paroreomyza montana* | Maui 'Alauahio | Fringillidae | *Plasmodium relictum*  Avipoxvirus | Hawaiian Islands (USA) | 2016 | EN | | (IUCN 2020) | |
| *Pelecanus philippensis* | Spot-billed Pelican | Pelecanidae | Avian Influenza virus (H5N1 subtype) | Southeast Asia | 2017 | NT | (IUCN 2020) | |  |
| *Pezoporus occidentalis* | Night Parrot | Psittacidae | Beak and Feather Disease  Avipoxvirus | Australia | 2019 | EN | (IUCN 2020) | |  |
| *Philesturnus carunculatus* | South Island Saddleback | Callaeidae | *Plasmodium relictum*  Avipoxvirus | New Zealand | 2016 | NT | (IUCN 2020) | |  |
| *Phoebastria irrorata* | Waved Albatross | Procellariiformes | Culicidae  Avipoxvirus | South America | 2018 | CR | | (IUCN 2020) |  |
| *Pica nutalli* | Yellow-billed Magpie | Corvidae | West Nile virus | California, USA | 2018 | VU | | (IUCN 2020) |  |
| *Poicephalus robustus* | Cape Parrot | Psittacidae | Beak and Feather disease | South Africa | 2017 | VU | | (IUCN 2020) |  |
| *Progne modesta* | Galapagos Martin | Hirundinidae | *Plasmodium relictum*  Avipoxvirus  *Philornis downi*  West Nile virus  Avian Influenza | Galápagos islands (Ecuador) | 2016 | EN | | (IUCN 2020) |  |

| **Specific epithet** | **Common Name** | **Family** | **Pathogen** | **Geographic Range** | **Assessment Year** | **IUCN Status** | **Citation** |
| --- | --- | --- | --- | --- | --- | --- | --- |
| *Psephotus pulcherrimus* | Paradise Parrot | Psittacidae | Unspecified | Eastern Australia | 1928, 2016 | EX | (Smith, Sax et al. 2006, IUCN 2020) |
| *Pseudonestor xanthophrys* | Maui Parrotbill | Fringillidae | *Plasmodium relictum*  Avipoxvirus | Hawaiian Islands (USA) | 2016 | CR | (IUCN 2020) |
| *Psittacula eques* | Echo Parakeet | Psittacidae | Beak and Feather Disease | Mauritius | 2019 | VU | (IUCN 2020) |
| *Psittirostra psittacea* | ʻōʻū | Fringillidae | *Plasmodium relictum* Avipoxvirus | Hawaiian Islands (USA) | 2018 | CR | (IUCN 2020) |
| *Pyrocephalus nanus* | Little Vermillion Flycatcher | Tyrannidae | Unspecified | Galápagos islands, Ecuador | 2017 | VU | (IUCN 2020) |
| *Rhabdotorrhinus exarhatus* | Sulawesi Hornbill | Bucerotidae | Unspecified | Indonesia | 2017 | VU | (IUCN 2020) |
| *Rhyticeros narcondami* | Narcondam Hornbill | Bucerotidae | Unspecified | India | 2017 | EN | (IUCN 2020) |
| *Rissa tridactyla* | Black-legged Kittiwake | Laridae | Unspecified | Global | 2019 | VU | (IUCN 2020) |
| *Setophaga chrysoparia* | Golden-cheeked Warbler | Parulidae | Unspecified | Central North America | 2018 | EN | (IUCN 2020) |
| *Somateria fischeri* | Spectacled Eider | Anatidae | Avian Influenza | Eastern Russia, Alaska (USA) | 2018 | NT | (IUCN 2020) |
| *Somateria mollissima* | Common Eider | Anatidae | Wellfleet Bay virus | Europe, North America, Eastern Asia | 2018 | NT | (IUCN 2020) |
| *Spheniscus magellanicus* | Magellanic Penguine | Spheniscidae | Unspecified | South America | 2018 | NT | (IUCN 2020) |
| *Strix occidentalis* | Spotted Owl | Strigidae | *Plasmodium relictum*  West Nile virus | Western United States | 2017 | NT | (IUCN 2020) |
| *Thalassarche cauta* | Shy Albatross | Diomedeidae | Avipoxvirus | Southern Hemisphere | 2018 | NT | (IUCN 2020) |
| *Vanellus vanellus* | Northern Lapwing | Charadriidae | Avian Botulism | Europe, Asia, Northern Africa | 2017 | NT | (IUCN 2020) |

**References**

IUCN. (2020). "The IUCN Red List of Threatened Species. Version 2020-1."

Savage, A. F. and E. C. Greiner (2004). "Hematozoa of the avian family Brachypteraciidae (the ground-rollers)." Journal of Parasitology **90**(6): 1468-1472.

Smith, K. F., D. F. Sax and K. D. Lafferty (2006). "Evidence for the role of infectious disease in species extinction and endangerment." Conservation Biology **20**(5): 1349-1357.
