# Supplementary material for: A Review of Pathogen Transmission at the Backyard Chicken–Wild Bird Interface
Source: Front Vet Sci. 2020 Sep 24;7:539925. doi: 10.3389/fvets.2020.539925 (PMC7541960; doi:10.3389/fvets.2020.539925)
Supplement: Supplementary file 2 [file Table_2.DOCX]

Supplemental Table 2. Tabulated below are pathogens that are either shared, or with the potential to be shared, with both backyard chickens and wild birds; but in which insufficient data to suggest spillover events were available in the literature.

| **Pathogen** | **Pathogen Type** | **Locations where antibodies or pathogen has been isolated from backyard chickens** | **Potential for spillover from backyard flocks** | **Susceptible Wild Birds (Host Range)** | **Mechanism of Transmission** | **Citations** |
| --- | --- | --- | --- | --- | --- | --- |
| Avian pathogenic *Escherichia coli* (APEC) | Bacteria | Near global, recently, it has been isolated from backyard chickens in Brazil, Gambia, Central Ethiopia, and India | High; due to wide host range of *E. coli* | European Starlings (*Sturnus vulgaris*) | Fecal-oral | (1-7). |
| *Mycobacterium avium* | Bacteria | Near global, cases from backyard chickens have been reported from nations such as Tunisia, the Czech Republic, Finland, Canada, the USA, and Ethiopia. | High, due to nearly global prevalence and multiple routes of transmission | Psittacines, passerines, raptors, gulls, waterfowl, Columbids, and a Common Snipe (*Gallinago gallinago*) (7-11) | Environmental, respiratory, fecal-oral, consumption of infectious carcasses or offal | (7-17) |
| *Salmonella enterica* serovars *Enteritidis, Infantis,* and *Typhimurium* are the most commonly reported in backyard chickens | Bacteria | Global; *Salmonella Enteritidis, Salmonella Infantis,* and *Salmonella Typhimurium* are the most commonly reported serovars in backyard chickens. | Very High; due to wide host range of serovars, and nearly global prevalence | Raptors, waterbirds, passerines, penguins and White Ibis (*Eudocimus albus*) | Fecal-oral | (18-28) |

| **Pathogen** | **Pathogen Type** | **Locations where antibodies or pathogen has been isolated from backyard chickens** | **Potential for spillover from backyard flocks** | **Susceptible Wild Birds (Host Range)** | **Mechanism of Transmission** | **Citations** |
| --- | --- | --- | --- | --- | --- | --- |
| *Campylobacter coli* | Bacteria | Near global, for example, it has been isolated from backyard chickens in New Zealand, the West Indies, Ecuador, and Kenya. | Likely, due to extremely wide host range | Multiple orders of birds notably in passerines and near passerines, Galliformes, waterfowl, raptors, waterbirds, Columbids, and gulls. | Fecal-oral, consumption of infectious carcasses or offal | (29-39). |
| *Campylobacter jejuni* | Bacteria | Near global, recently it has been isolated from backyard chickens in Finland, New Zealand, Ecuador, Kenya, the West Indies, and Nigeria. | Spillover potential is unlikely due to host specificity of strains | N/A | Fecal-oral, consumption of infectious carcasses or offal | (21, 29-32, 40-42) |
| *Avibacterium paragallinarum* | Bacteria | Near global, for example, it has been isolated from backyard flocks in India, the USA, Ethiopia, the UK, and Vietnam. | Spillover potential is unlikely , only one report found in wild birds | Raptors | Aerosols, fomites | (43-48) |
| *Ornithobacterium rhinotracheale* | Bacteria | First identified in the 1990’s, since then it has been isolated on a nearly global level, including backyard flocks in Belgium, New Zealand, Hungary, the USA, and Vietnam. | Poorly understood, although a degree of host specificity reported | Columbids, raptors, near passerines and Red-wattled Lapwings *(Vanellus indicus)* | Aerosols, fomites, vertical transmission | (47, 49-54) |
| *Erysipelothrix rhusiopathiae* | Bacteria | No prevalence studies in backyard chickens found | Poorly understood due to complex transmission route and lack of data on backyard chickens | Passerines, waterfowl, Galliformes, and an Emu (*Dromaius novaehollandiae*) | Vector-borne | (55-57) |

| **Pathogen** | **Pathogen Type** | **Locations where antibodies or pathogen has been isolated from backyard chickens** | **Potential for spillover from backyard flocks** | **Susceptible Wild Birds (Host Range)** | **Mechanism of Transmission** | **Citations** |
| --- | --- | --- | --- | --- | --- | --- |
| *Clostridium botulism* | Bacteria | Few studies in backyard chickens available. One was recently performed in Brazil, while the other was performed in Korea. | Poorly understood due to complex transmission route and limited data in backyard chickens | Types E and C frequently associated with waterfowl and associated aquatic birds. Highland guans (*Penelopina nigra*) have also been affected | Ingestion of endotoxin | (58-62) |
| *Listeria*  *monocytogenes* | Bacteria | Few studies in backyard chickens available. The available literature are from Finland, the USA, and Nigeria | Poorly understood as the bacterium is rarely isolated from live chickens | Multiple species affected, e.g. gulls, passerines and near passerines raptors, and Galliformes | Aerosols, ingestion | (21, 63-67) |
| *Klebsiella pneumoniae* | Bacteria | Likely has a nearly global distribution, but studies seem to be more prevalent in southeast Asia for the backyard sector. Recent reports have come from Pakistan, Nigeria, Cambodia, India, and Nepal | Likely, due to its wide host range | Waterfowl, gulls, passerines, near passerines, psittacines | Fomites | (68-77) |
| *Chlamydia psittaci* | Bacteria | Likely has a worldwide distribution, but studies tend to focus on commercial operations. Backyard chicken studies have been conducted in Italy, Germany, Croatia, and Nigeria | Poorly understood due to limited data in backyard chickens | Multiple species, e.g. Columbiformes, Galliformes, raptors, psittacines, passerines, and Flightless Cormorants (*Phalacrocorax harrisi*) | Aerosols, fecal-oral | (78-86) |

| **Pathogen** | **Pathogen Type** | **Locations where antibodies or pathogen has been isolated from backyard chickens** | **Potential for spillover from backyard flocks** | **Susceptible Wild Birds (Host Range)** | **Mechanism of Transmission** | **Citations** |
| --- | --- | --- | --- | --- | --- | --- |
| *Chlamydia  gallinacea* | Bacteria | Recently identified in the late 2010’s, thus the literature is limited on the agent. Studies have since been performed in Mexico, Italy, and Australia | Poorly understood given that the bacterium was recently identified | Psittacines and Galliformes | Fecal-oral, vertical transmission suspected | (80, 87-89) |
| *Aspergillus flavus* | Fungus | Nearly global in distribution. Studies in backyard flocks have been recently performed in Iran, Costa Rica, Egypt, Bangladesh, and Italy | Likely high, although the environmental route of transmission is more likely | Multiple species, e.g. gulls, raptors, Columbids, Galliformes, psittacines, penguins, passerines | Ingestion, Environmental, Aerosol | (90-96) |
| *Aspergillus fumigatus* | Fungus | Nearly global in distribution, however most studies consider commercial operations. Recent studies have been conducted in Costa Rica, Bangladesh, and the USA | Likely high, although the environmental route of transmission is more likely | Multiple species, e.g., raptors, waterfowl, gulls, Galliformes, penguins, and passerines | Ingestion, Environmental, Aerosol | (16, 91, 93, 97-101) |
| *Candida albicans* | Fungus | Nearly global in distribution, however most studies consider commercial operations. Studies on backyard chickens involving this fungus have been performed in the USA, Japan, Nigeria, and Egypt | Likely high, although the environmental route of transmission is more likely | Multiple species, e.g. gulls, cormorants. penguins, passerines, waterfowl, raptors, Columbiformes | Ingestion, Environmental, Aerosol | (16, 102-106) |

| **Pathogen** | **Pathogen Type** | **Locations where antibodies or pathogen has been isolated from backyard chickens** | **Potential for spillover from backyard flocks** | **Susceptible Wild Birds (Host Range)** | **Mechanism of Transmission** | **Citations** |
| --- | --- | --- | --- | --- | --- | --- |
| Avian bornavirus | Virus | No studies concerning avian bornavirus in backyard chickens found. | Spillover potential is unlikely due to host specificity of virus | Waterfowl and psittacines | Fecal-oral, vertical transmission, | (107, 108) |
| Infectious laryngotracheitis | Virus | Near global distribution, for example, it has been recently isolated from backyard chickens in Brazil, Canada, the USA, Ethiopia, Costa Rica, and Thailand | Spillover potential is unlikely due to host specificity of virus | N/A | Aerosols, fomites, bird-bird contact | (91, 109-114) |
| Avian hepatitis E virus | Virus | Likely widespread, although most studies consider commercial operations. An outbreak in backyard chickens was reported in the USA | Poorly understood due to a lack of data in backyard flocks, however a spillover event was suspected in 2017 | Columbids, raptors, waterbirds, and passerines | Fecal-oral | (115-117) |
| Avian metapneumovirus | Virus | Nearly global in distribution. Studies in backyard flocks have been recently performed in Oman, Brazil, Belgium, and Kazakhstan, and Bulgaria | Likely, due to extremely wide host range | Multiple species, e.g. aquatic birds and waterfowl, Columbids, Craciformes, psittacines, raptors | Aerosols, fomites, bird-bird contact | (49, 118-125) |

| **Pathogen** | **Pathogen Type** | **Locations where antibodies or pathogen has been isolated from backyard chickens** | **Potential for spillover from backyard flocks** | **Susceptible Wild Birds (Host Range)** | **Mechanism of Transmission** | **Citations** |
| --- | --- | --- | --- | --- | --- | --- |
| Avian nephritis virus | Virus | Poorly studied, only one study found concerning backyard chickens in Nigeria | Poorly understood due to a lack of data in backyard flocks and wild birds | Columbiformes, Pelecaniformes, Charadriiformes | Fecal-oral, vertical transmission | (126-129) |
| Chicken Anemia Virus | Virus | Nearly global, recently reported from Brazil, Costa Rica, Nigeria, Japan, and Bulgaria | Spillover potential is unlikely; lack of wild bird data | N/A | Fecal-oral, fomites, aerosol,  vertical transmission | (91, 130-134) |
| Lymphoproliferative disease virus | Virus | No prevalence studies in backyard turkeys found | Poorly understood; lack of data in backyard flocks | Wild Turkeys (*Meleagris gallipavo*) | Horizontal transmission suspected | (135, 136) |
| Gapeworm  (*Syngamus trachea* ) | Parasite | Nearly global distribution, having recently reported in backyard chickens from the USA, Nigeria, Uganda, and Nepal | Very High; due to wide host range of parasite, and nearly global prevalence | Multiple species, e.g. Galliformes, waterfowl, passerines, near passerines, and Gruiformes | Fecal-oral | (137-144) |
| *Heterakis gallinarium* | Parasite | Nearly global, recently reported from India, Brazil, Iran, Denmark, Iran, the West Indies, and Bangladesh | Likely high, although more data on the prevalence of the parasite in wild birds is needed | Columbids, Galliformes | Fecal-oral | (134, 145-152) |

**References**

1. E. Oliveira, M. Cardozo, M. Borzi, C. Borges, E. Guastalli and F. Ávila: Highly Pathogenic and Multidrug Resistant Avian Pathogenic Escherichia Coli in Free-Range Chickens from Brazil. *Brazilian Journal of Poultry Science*, 21 (2019)

2. C. A. Borges, N. J. Tarlton and L. W. Riley: Escherichia coli from Commercial Broiler and Backyard Chickens Share Sequence Types, Antimicrobial Resistance Profiles, and Resistance Genes with Human Extraintestinal Pathogenic Escherichia coli. *Foodborne Pathogens and Disease*, 16(12), 813-822 (2019) doi:10.1089/fpd.2019.2680

3. E. Foster-Nyarko, N.-F. Alikhan, A. Ravi, N. M. Thomson, S. Jarju, B. A. Kwambana-Adams, A. Secka, J. O’Grady, M. Antonio and M. J. Pallen: Genomic diversity of <em>Escherichia coli</em> isolates from backyard chickens and guinea fowl in the Gambia. *bioRxiv*, 2020.05.14.096289 (2020) doi:10.1101/2020.05.14.096289

4. E. J. Sarba, K. A. Kelbesa, M. D. Bayu, E. Z. Gebremedhin, B. M. Borena and A. Teshale: Identification and antimicrobial susceptibility profile of Escherichia coli isolated from backyard chicken in and around ambo, Central Ethiopia. *BMC Veterinary Research*, 15(1), 85 (2019) doi:10.1186/s12917-019-1830-z

5. I. Samanta, S. N. Joardar, P. K. Das, P. Das, T. K. Sar, T. K. Dutta, S. Bandyopadhyay, S. Batabyal and D. P. Isore: Virulence repertoire, characterization, and antibiotic resistance pattern analysis of Escherichia coli isolated from backyard layers and their environment in India. *Avian diseases*, 58(1), 39-45 (2014)

6. S. Boro, D. Pathak, G. Saikia and M. Buragohain: Prevalence of Colibacillosis in birds in and around Guwahati city (Assam). *Journal of Entomology and Zoology Studies*, 6(1), 1000-1003 (2018)

7. S. M. Gaukler, G. M. Linz, J. S. Sherwood, N. W. Dyer, W. J. Bleier, Y. M. Wannemuehler, L. K. Nolan and C. M. Logue: Escherichia coli, Salmonella, and Mycobacterium avium subsp. paratuberculosis in Wild European Starlings at a Kansas Cattle Feedlot. *Avian Diseases*, 53(4), 544-551 (2009) doi:10.1637/8920-050809-Reg.1

8. J. L. Corn, E. J. B. Manning, S. Sreevatsan and J. R. Fischer: Isolation of Mycobacterium avium subsp. paratuberculosis from Free-Ranging Birds and Mammals on Livestock Premises. *Applied and Environmental Microbiology*, 71(11), 6963-6967 (2005) doi:10.1128/aem.71.11.6963-6967.2005

9. T. Smit, A. Eger, J. Haagsma and T. Bakhuizen: Avian tuberculosis in wild birds in the Netherlands. *Journal of Wildlife Diseases*, 23(3), 485-487 (1987) doi:10.7589/0090-3558-23.3.485

10. P. Gronesova, M. Ficova, A. Mizakova, P. Kabat, A. Trnka and T. Betakova: Prevalence of avian influenza viruses, Borrelia garinii, Mycobacterium avium, and Mycobacterium avium subsp. paratuberculosis in waterfowl and terrestrial birds in Slovakia, 2006. *Avian Pathology*, 37(5), 537-543 (2008) doi:10.1080/03079450802356953

11. S. N. Godoy, S. M. Sakamoto, C. D. d. Paula, J. L. Catão-Dias and E. R. Matushima: Detection of Mycobacterium avium in pet birds. *Brazilian Journal of Microbiology*, 40, 265-268 (2009)

12. K. Kaboudi, A. Amara and M. Bouzouaia: Avian tuberculosis in a backyard poultry flock in Tunisia: Case report. *International Journal of Veterinary Sciences and Animal Husbandry*, 2, 34-37 (2017)

13. J. E. Shitaye, L. Matlova, A. Horvathova, M. Moravkova, L. Dvorska-Bartosova, F. Treml, J. Lamka and I. Pavlik: Mycobacterium avium subsp. avium distribution studied in a naturally infected hen flock and in the environment by culture, serotyping and IS901 RFLP methods. *Veterinary Microbiology*, 127(1), 155-164 (2008) doi:<https://doi.org/10.1016/j.vetmic.2007.07.026>

14. L. Pohjola, L. Rossow, A. Huovilainen, T. Soveri, M.-L. Hänninen and M. Fredriksson-Ahomaa: Questionnaire study and postmortem findings in backyard chicken flocks in Finland. *Acta Veterinaria Scandinavica*, 57(1), 57-72 (2015) doi:10.1186/s13028-015-0095-1

15. A. A. Mutalib and C. Riddell: Epizootiology and pathology of avian tuberculosis in chickens in Saskatchewan. *The Canadian Veterinary Journal*, 29(10), 840 (1988)

16. K. J. Cadmus, A. Mete, M. Harris, D. Anderson, S. Davison, Y. Sato, J. Helm, L. Boger, J. Odani, M. D. Ficken and K. L. Pabilonia: Causes of mortality in backyard poultry in eight states in the United States. *Journal of Veterinary Diagnostic Investigation*, 31(3), 318-326 (2019) doi:10.1177/1040638719848718

17. A. Kindu and G. Getaneh: Prevalence of Avian Tuberculosis in Domestic Chickens in Selected Sites of Ethiopia. *J Vet Sci Technol*, 7(377), 2 (2016)

18. R. Jafari, M. Ghorbanpour and A. Jaideri: An investigation into Salmonella infection status in backyard chickens in Iran. *International Journal of Poultry Science*, 6(3), 227-229 (2007)

19. C. S. Emadi, M. Hasanzadeh, M. M. Bozorg and S. Mirzaei: Characterization of the Salmonella isolates from backyard chickens in north of Iran, by serotyping, multiplex PCR and antibiotic resistance analysis. *Archives of Razi Institute*, 64(2) (2009)

20. A. Mete, F. Giannitti, B. Barr, L. Woods and M. Anderson: Causes of Mortality in Backyard Chickens in Northern California: 2007–2011. *Avian Diseases*, 57(2), 311-315 (2013) doi:10.1637/10382-092312-Case.1

21. L. Pohjola, S. Nykäsenoja, R. Kivistö, T. Soveri, A. Huovilainen, M. L. Hänninen and M. Fredriksson-Ahomaa: Zoonotic Public Health Hazards in Backyard Chickens. *Zoonoses and Public Health*, 63(5), 420-430 (2016) doi:10.1111/zph.12247

22. R. S. Hendriksen, A. R. Vieira, S. Karlsmose, D. M. Lo Fo Wong, A. B. Jensen, H. C. Wegener and F. M. Aarestrup: Global Monitoring of Salmonella Serovar Distribution from the World Health Organization Global Foodborne Infections Network Country Data Bank: Results of Quality Assured Laboratories from 2001 to 2007. *Foodborne Pathogens and Disease*, 8(8), 887-900 (2011) doi:10.1089/fpd.2010.0787

23. J. Millán, G. Aduriz, B. Moreno, R. Juste and M. Barral: Salmonella isolates from wild birds and mammals in the Basque Country (Spain). *Revue Scientifique et Technique-Office International des Epizooties*, 23(3), 905-912 (2004)

24. O. Obukhovska: The Natural Reservoirs of Salmonella Enteritidis in Populations of Wild Birds. *Online Journal of Public Health Informatics*, 5(1) (2013) doi:10.5210/ojphi.v5i1.4569

25. H. Palmgren, M. Sellin, S. Bergström and B. Olsen: Enteropathogenic Bacteria in Migrating Birds Arriving in Sweden. *Scandinavian Journal of Infectious Diseases*, 29(6), 565-568 (1997) doi:10.3109/00365549709035895

26. T. W. Pennycott, A. Park and H. A. Mather: Isolation of different serovars of <em>Salmonella enterica</em> from wild birds in Great Britain between 1995 and 2003. *Veterinary Record*, 158(24), 817-820 (2006) doi:10.1136/vr.158.24.817

27. H. Palmgren, D. McCafferty, A. AspÁN, T. Broman, M. Sellin, R. Wollin, S. BergstrÖM and B. Olsen: Salmonella in sub-Antarctica: low heterogeneity in salmonella serotypes in South Georgian seals and birds. *Epidemiology and Infection*, 125(2), 257-262 (2001) doi:10.1017/S0950268899004586

28. S. M. Hernandez, C. N. Welch, V. E. Peters, E. K. Lipp, S. Curry, M. J. Yabsley, S. Sanchez, A. Presotto, P. Gerner-Smidt, K. B. Hise, E. Hammond, W. M. Kistler, M. Madden, A. L. Conway, T. Kwan and J. J. Maurer: Urbanized White Ibises (Eudocimus albus) as Carriers of Salmonella enterica of Significance to Public Health and Wildlife. *PLOS ONE*, 11(10), e0164402 (2016) doi:10.1371/journal.pone.0164402

29. J. Anderson, B. J. Horn and B. J. Gilpin: The Prevalence and Genetic Diversity of Campylobacter spp. in Domestic ‘Backyard’ Poultry in Canterbury, New Zealand. *Zoonoses and Public Health*, 59(1), 52-60 (2012) doi:10.1111/j.1863-2378.2011.01418.x

30. R. Sharma, K. Tiwari, V. M. Belmar, S. Kumar, S. M. Goyal, V. A. Amadi, N. Watson and H. Hariharan: Prevalence and antimicrobial resistance of Campylobacter species isolated from backyard chickens in Grenada, West Indies. *Microbiology Research Journal International*, 1-8 (2016)

31. S. Ochoa, R. J. Simaluiza, Z. Toledo and H. Fernández: Frequency and antimicrobial behaviour of thermophilic Campylobacter species isolated from Ecuadorian backyard chickens. *Archivos de Medicina Veterinaria*, 48(3), 311-314 (2016)

32. T. N. M. Nguyen, H. Hotzel, J. Njeru, J. Mwituria, H. El-Adawy, H. Tomaso, H. Neubauer and H. M. Hafez: Antimicrobial resistance of Campylobacter isolates from small scale and backyard chicken in Kenya. *Gut Pathogens*, 8(1), 39 (2016) doi:10.1186/s13099-016-0121-5

33. C. Varga, M. T. Guerin, M. L. Brash, D. Slavic, P. Boerlin and L. Susta: Antimicrobial resistance in Campylobacter jejuni and Campylobacter coli isolated from small poultry flocks in Ontario, Canada: A two-year surveillance study. *PLOS ONE*, 14(8), e0221429 (2019) doi:10.1371/journal.pone.0221429

34. B. Hald, M. N. Skov, E. M. Nielsen, C. Rahbek, J. J. Madsen, M. Wainø, M. Chriél, S. Nordentoft, D. L. Baggesen and M. Madsen: Campylobacter jejuni and Campylobacter coli in wild birds on Danish livestock farms. *Acta Veterinaria Scandinavica*, 58(1), 11 (2016) doi:10.1186/s13028-016-0192-9

35. O. Rosef, G. Kapperud, S. Lauwers and B. Gondrosen: Serotyping of Campylobacter jejuni, Campylobacter coli, and Campylobacter laridis from domestic and wild animals. *Applied and Environmental Microbiology*, 49(6), 1507-1510 (1985)

36. Robino, Tomassone, Tramuta, Rodo, Giammarino, Vaschetti and Nebbia: Prevalence of Campylobacter jejuni, Campylobacter coli and enteric Helicobacter in domestic and free living birds in North-Western Italy. *Schweizer Archiv für Tierheilkunde*, 152(9), 425-431 (2010) doi:10.1024/0036-7281/a000094

37. L. A. Hughes, M. Bennett, P. Coffey, J. Elliott, T. R. Jones, R. C. Jones, A. Lahuerta-Marin, A. H. Leatherbarrow, K. McNiffe, D. Norman, N. J. Williams and J. Chantrey: Molecular Epidemiology and Characterization of <em>Campylobacter</em> spp. Isolated from Wild Bird Populations in Northern England. *Applied and Environmental Microbiology*, 75(10), 3007-3015 (2009) doi:10.1128/aem.02458-08

38. J. I. Keller, W. G. Shriver, J. Waldenstrom, P. Griekspoor and B. Olsen: Prevalence of Campylobacter in Wild Birds of the Mid-Atlantic Region, USA. *Journal of Wildlife Diseases*, 47(3), 750-754 (2011) doi:10.7589/0090-3558-47.3.750

39. H. Fernández, W. Gesche, A. Montefusco and R. Schlatter: Wild birds as reservoir of thermophilic enteropathogenic Campylobacter species in southern Chile. *Memórias do Instituto Oswaldo Cruz*, 91, 699-700 (1996)

40. P. Griekspoor, F. M. Colles, N. D. McCarthy, P. M. Hansbro, C. Ashhurst‐Smith, B. Olsen, D. Hasselquist, M. C. Maiden and J. Waldenström: Marked host specificity and lack of phylogeographic population structure of Campylobacter jejuni in wild birds. *Molecular ecology*, 22(5), 1463-1472 (2013) doi:10.1111/mec.12144

41. M. C. Brena, Y. Mekonnen, J. M. Bettridge, N. J. Williams, P. Wigley, T. Sisay Tessema and R. M. Christley: Changing risk of environmental Campylobacter exposure with emerging poultry production systems in Ethiopia. *Epidemiology and Infection*, 144(3), 567-575 (2015) doi:10.1017/S0950268815001429

42. J. O. Adekeye, P. A. Abdu and E. K. Bawa: Campylobacter fetus Subsp. jejuni in Poultry Reared under Different Management Systems in Nigeria. *Avian Diseases*, 33(4), 801-803 (1989) doi:10.2307/1591163

43. T. M. N. Muhammad and B. Sreedevi: Detection of Avibacterium paragallinarum by Polymerase chain reaction from outbreaks of Infectious coryza of poultry in Andhra Pradesh. *Veterinary world*, 8(1), 103-108 (2015) doi:10.14202/vetworld.2015.103-108

44. K. A. Clothier, A. Torain and S. Reinl: Surveillance for Avibacterium paragallinarum in autopsy cases of birds from small chicken flocks using a real-time PCR assay. *Journal of Veterinary Diagnostic Investigation*, 31(3), 364-367 (2019) doi:10.1177/1040638719844297

45. I. Dereja and D. Hailemichael: Infectious Coryza in Jimma Backyard Chicken Farms: Clinical and Bacteriological Investigation. *J Vet Sci Technol*, 8(412), 2 (2017)

46. D. d. B. Welchman, S. A. King, P. Wragg, A. M. Wood, R. M. Irvine, W. J. Pepper, R. Dijkman and J. J. de Wit: Infectious coryza in chickens in Great Britain. *Veterinary Record*, 167(23), 912-913 (2010) doi:10.1136/vr.c6841

47. N. T. B. Van, N. T. P. Yen, N. T. Nhung, N. V. Cuong, B. T. Kiet, N. V. Hoang, V. B. Hien, N. Chansiripornchai, M. Choisy, A. Ribas, J. Campbell, G. Thwaites and J. Carrique-Mas: Characterization of viral, bacterial, and parasitic causes of disease in small-scale chicken flocks in the Mekong Delta of Vietnam. *Poultry Science*, 99(2), 783-790 (2020) doi:<https://doi.org/10.1016/j.psj.2019.10.033>

48. M. Bezjian and G. V. Kollias: American Kestrel (Falco spaverius) Fledgling With Severe Bilateral Periorbital Swelling and Infection With Mycoplasma buteonis, Avibacterium (Pasteurella) gallinarum, and Staphylococcus pasteuri. *Journal of Avian Medicine and Surgery*, 28(2), 127-131 (2014)

49. R. Haesendonck, M. Verlinden, G. Devos, T. Michiels, P. Butaye, F. Haesebrouck, F. Pasmans and A. Martel: High Seroprevalence of Respiratory Pathogens in Hobby Poultry. *Avian Diseases*, 58(4), 623-627 (2014)

50. H. J. Ha, N. Christensen, S. Humphrey, T. Haydon, G. Bernardi and T. Rawdon: The First Detection of Ornithobacterium rhinotracheale in New Zealand. *Avian Diseases*, 60(4), 856-859 (2016) doi:10.1637/11457-062116-Case

51. R. Szabó, E. Wehmann, L. Makrai, C. Nemes, É. Gyuris, Á. Thuma and T. Magyar: Characterization of Ornithobacterium rhinotracheale field isolates from Hungary. *Avian Pathology*, 46(5), 506-514 (2017) doi:10.1080/03079457.2017.1321104

52. T. Derksen, R. Lampron, R. Hauck, M. Pitesky and R. A. Gallardo: Biosecurity Assessment and Seroprevalence of Respiratory Diseases in Backyard Poultry Flocks Located Close to and Far from Commercial Premises. *Avian Diseases*, 62(1), 1-5 (2018) doi:10.1637/11672-050917-Reg.1

53. S. Réka, W. Enikő and M. Tibor: Antimicrobial susceptibility of Bordetella Avium and Ornithobacterium Rhinotracheale strains from wild and domesticated birds in Hungary. *Acta Veterinaria Hungarica*, 63(4), 413-424 (2015) doi:10.1556/004.2015.039

54. S. Thieme, H. M. Hafez, S. Gutzer, N. Warkentin, D. Lüschow and K. Mühldorfer: Multilocus sequence typing of Ornithobacterium rhinotracheale isolated from pigeons and birds of prey revealed new insights into its population structure. *Veterinary and Animal Science*, 1-2, 15-20 (2016) doi:<https://doi.org/10.1016/j.vas.2016.10.002>

55. H. Eriksson, E. Bagge, V. Båverud, C. Fellström and D. S. Jansson: Erysipelothrix rhusiopathiae contamination in the poultry house environment during erysipelas outbreaks in organic laying hen flocks. *Avian Pathology*, 43(3), 231-237 (2014) doi:10.1080/03079457.2014.907485

56. G. J. Eamens, M. J. Turner and R. E. Catt: Serotypes of Erysipelothrix rhusiopathiae in Australian pigs, small ruminants, poultry, and captive wild birds and animals. *Australian Veterinary Journal*, 65(8), 249-252 (1988) doi:10.1111/j.1751-0813.1988.tb14311.x

57. M. J. Morgan, J. O. Britt, J. M. Cockrill and M. L. Eiten: Erysipelothrix Rhusiopathiae Infection in an Emu (Dromaius Novaehollandiae). *Journal of Veterinary Diagnostic Investigation*, 6(3), 378-379 (1994) doi:10.1177/104063879400600319

58. G. E. Hannett, W. B. Stone, S. W. Davis and D. Wroblewski: Biodiversity of <em>Clostridium botulinum</em> Type E Associated with a Large Outbreak of Botulism in Wildlife from Lake Erie and Lake Ontario. *Applied and Environmental Microbiology*, 77(3), 1061-1068 (2011) doi:10.1128/aem.01578-10

59. G.-H. Woo, H.-Y. Kim, Y.-C. Bae, Y. H. Jean, S.-S. Yoon, E.-J. Bak, E. K. Hwang and Y.-S. Joo: Outbreak of botulism (Clostridium botulinum type C) in wild waterfowl: Seoul, Korea. *Journal of wildlife diseases*, 46(3), 951-955 (2010)

60. R. O. S. Silva, R. A. Martins, R. A. Assis, C. A. Oliveira Junior and F. C. F. Lobato: Type C botulism in domestic chickens, dogs and black-pencilled marmoset (Callithrix penicillata) in Minas Gerais, Brazil. *Anaerobe*, 51, 47-49 (2018) doi:<https://doi.org/10.1016/j.anaerobe.2018.03.013>

61. I. Jang, M. S. Kang, H. R. Kim, J. Y. Oh, J. I. Lee, H. S. Lee and Y. K. Kwon: Occurrence of Avian Botulism in Korea During the Period from June to September 2012. *Avian Diseases*, 58(4), 666-669 (2014) doi:10.1637/10793-020414-Case

62. R. O. S. Silva, S. Y. M. Gómez, L. B. Medeiros, M. V. R. Marques, A. S. G. Silva, E. N. Mureb, C. A. Oliveira Junior, S. M. Favoretto, F. C. F. Lobato and N. R. S. Martins: Antitoxin therapy of natural avian botulism outbreaks occurred in Brazil. *Anaerobe*, 48, 115-117 (2017) doi:<https://doi.org/10.1016/j.anaerobe.2017.08.005>

63. R. Crespo, M. M. Garner, S. G. Hopkins and D. H. Shah: Outbreak of Listeria monocytogenes in an urban poultry flock. *BMC Veterinary Research*, 9(1), 204 (2013) doi:10.1186/1746-6148-9-204

64. O. O. Oni, A. A. Adesiyun, J. O. Adekeye and S. N. A. Sai'du: Sero-prevalence of agglutinins to Listeria monocytogenes in Nigerian domestic animals. *Bacteriolgie*, 42(3), 383-388 (1989)

65. D. R. Fenlon: Wild birds and silage as reservoirs of Listeria in the agricultural environment. *Journal of Applied Bacteriology*, 59(6), 537-543 (1985) doi:10.1111/j.1365-2672.1985.tb03357.x

66. S. Hellström, K. Kiviniemi, T. Autio and H. Korkeala: Listeria monocytogenes is common in wild birds in Helsinki region and genotypes are frequently similar with those found along the food chain. *Journal of Applied Microbiology*, 104(3), 883-888 (2008) doi:10.1111/j.1365-2672.2007.03604.x

67. J. M. Hatkin, W. E. Phillips Jr and G. A. Hurst: Isolation of Listeria monocytogenes from an eastern wild turkey. *Journal of Wildlife Diseases*, 22(1), 110-112 (1986)

68. M. Shoaib, A. Kamboh, A. Sajid, G. Mughal, R. Leghari, K. Malhi, S. Bughio, A. Ali, S. Alam and S. Khan: Prevalence of extended spectrum beta-lactamase producing Enterobacteriaceae in commercial broilers and backyard chickens. *Adv. Anim. Vet. Sci*, 4(4), 209-214 (2016)

69. Y. Dashe, H. Kazeem, P. Abdu, M. Bello and M. Odugbo: Klebsiella pneumoniae isolated from birds affected by natural outbreaks of highly pathogenic avian influenza (H5N1) in Nigeria. *Sokoto Journal of Veterinary Sciences*, 7(2) (2008)

70. C. Atterby, K. Osbjer, V. Tepper, E. Rajala, J. Hernandez, S. Seng, D. Holl, J. Bonnedahl, S. Börjesson, U. Magnusson and J. D. Järhult: Carriage of carbapenemase- and extended-spectrum cephalosporinase-producing Escherichia coli and Klebsiella pneumoniae in humans and livestock in rural Cambodia; gender and age differences and detection of blaOXA-48 in humans. *Zoonoses and Public Health*, 66(6), 603-617 (2019) doi:10.1111/zph.12612

71. A. Mahanti, P. Ghosh, I. Samanta, S. N. Joardar, S. Bandyopadhyay, D. Bhattacharyya, J. Banerjee, S. Batabyal, T. K. Sar and T. K. Dutta: Prevalence of CTX-M-Producing Klebsiella spp. in Broiler, Kuroiler, and Indigenous Poultry in West Bengal State, India. *Microbial Drug Resistance*, 24(3), 299-306 (2018) doi:10.1089/mdr.2016.0096

72. S. Hosuru Subramanya, I. Bairy, N. Nayak, R. Amberpet, S. Padukone, Y. Metok, D. R. Bhatta and B. Sathian: Detection and characterization of ESBL-producing Enterobacteriaceae from the gut of healthy chickens, Gallus gallus domesticus in rural Nepal: Dominance of CTX-M-15-non-ST131 Escherichia coli clones. *PLOS ONE*, 15(5), e0227725 (2020) doi:10.1371/journal.pone.0227725

73. A. A. Kamboh, M. Shoaib, S. H. Abro, M. A. Khan, K. K. Malhi and S. Yu: Antimicrobial Resistance in Enterobacteriaceae Isolated from Liver of Commercial Broilers and Backyard Chickens. *Journal of Applied Poultry Research*, 27(4), 627-634 (2018) doi:<https://doi.org/10.3382/japr/pfy045>

74. S. Raza, M. Mohsin, W. A. Madni, F. Sarwar, M. Saqib and B. Aslam: First Report of bla CTX-M-15-Type ESBL-Producing Klebsiella pneumoniae in wild migratory birds in Pakistan. *Ecohealth*, 14(1), 182-186 (2017)

75. J. Bonnedahl, J. Hernandez, J. Stedt, J. Waldenström, B. Olsen and M. Drobni: Extended-spectrum β-lactamases in Escherichia coli and Klebsiella pneumoniae in Gulls, Alaska, USA. *Emerging infectious diseases*, 20(5), 897-899 (2014) doi:10.3201/eid2005.130325

76. Y. M. Davies, M. P. V. Cunha, M. G. X. Oliveira, M. C. V. Oliveira, N. Philadelpho, D. C. Romero, L. Milanelo, M. B. Guimarães, A. J. P. Ferreira, A. M. Moreno, L. R. M. Sá and T. Knöbl: Virulence and antimicrobial resistance of Klebsiella pneumoniae isolated from passerine and psittacine birds. *Avian Pathology*, 45(2), 194-201 (2016) doi:10.1080/03079457.2016.1142066

77. J. Stenkat, M.-E. Krautwald-Junghanns, A. Schmitz Ornés, A. Eilers and V. Schmidt: Aerobic cloacal and pharyngeal bacterial flora in six species of free-living birds. *Journal of Applied Microbiology*, 117(6), 1564-1571 (2014) doi:10.1111/jam.12636

78. E. K. Travis, F. H. Vargas, J. Merkel, N. Gottdenker, R. E. Miller and P. G. Parker: Hematology, plasma chemistry, and serology of the flightless cormorant (Phalacrocorax harrisi) in the Galapagos Islands, Ecuador. *Journal of Wildlife Diseases*, 42(1), 133-141 (2006)

79. M. Crispo, J. Blakey, H. Shivaprasad, K. Laroucau, F. Vorimore, R. Aaziz, A. Bickford, J. Pesavento and S. T. Stoute: Chlamydiosis in a Gouldian Finch (Erythrura gouldiae). *Avian diseases*, 64(2), 216-222 (2020)

80. M. Donati, K. Laroucau, A. Guerrini, A. Balboni, D. Salvatore, E. Catelli, C. Lupini, A. Levi and A. Di Francesco: Chlamydiosis in Backyard Chickens (Gallus gallus) in Italy. *Vector-Borne and Zoonotic Diseases*, 18(4), 222-225 (2018) doi:10.1089/vbz.2017.2211

81. D. H. Tomić, Ž. Gottstein, V. Savić, M. Tišljar, M. Lukač and E. Prukner-Radovčić: Chlamydia psittaci associated with pox virus infection in laying hen flock. In: *Simpozij Peradarski Dani 2013. S međunarodnim sudjelovanjem Hrvatska, Šibenik, 15.-18. Svibnja 2013.* Ed M. Balenović. Croatian Veterinary Institute, Poultry Centre, Zagreb (2013)

82. G. Jatau, C. Reuben, O. Chukwu and M. Bdliya: Serological and Histochemical Detection of Chlamydophila psittaci in Poultry at Live Bird Markets in Kaduna Metropolis, Nigeria. *Journal of Microbiology and Biomedical Research*, 2(4) (2016)

83. M. Donati, K. Laroucau, M. Delogu, F. Vorimore, R. Aaziz, E. Cremonini, R. Biondi, C. Cotti, R. Baldelli and A. Di Francesco: Chlamydia psittaci in Eurasian Collared Doves (Streptopelia decaocto) in Italy. *Journal of Wildlife Diseases*, 51(1), 214-217 (2015) doi:10.7589/2014-01-010

84. R. P. Maluping, R. B. Oronan and S. U. Toledo: Detection of Chlamydophila psittaci antibodies from captive birds at the Ninoy Aquino Parks and Wildlife Nature Center, Quezon City, Philippines. *Ann Agric Environ Med*, 14(1), 191-3 (2007)

85. H. Gerbermann and R. Korbel: The occurrence of Chlamydia psittaci infections in raptors from wildlife preserves. *Tierarztl Prax*, 21(3), 217-24 (1993)

86. W. Gaede, K.-F. Reckling, B. Dresenkamp, S. Kenklies, E. Schubert, U. Noack, H.-M. Irmscher, C. Ludwig, H. Hotzel and K. Sachse: Chlamydophila psittaci Infections in Humans during an Outbreak of Psittacosis from Poultry in Germany. *Zoonoses and Public Health*, 55(4), 184-188 (2008) doi:10.1111/j.1863-2378.2008.01108.x

87. E. Ornelas-Eusebio, G. Garcia-Espinosa, F. Vorimore, R. Aaziz, B. Durand, K. Laroucau and G. Zanella: Cross-sectional study on Chlamydiaceae prevalence and associated risk factors on commercial and backyard poultry farms in Mexico. *Preventive Veterinary Medicine*, 176, 104922 (2020) doi:<https://doi.org/10.1016/j.prevetmed.2020.104922>

88. H. Stokes, J. Martens, A. Chamings, K. Walder, M. Berg, Y. Segal and A. Bennett: Identification of Chlamydia gallinacea in a parrot and in free-range chickens in Australia. *Australian Veterinary Journal*, 97(10), 398-400 (2019) doi:10.1111/avj.12856

89. B. R. Vogler, M. Trinkler, H. Marti, N. Borel, T. Pesch, B. Prähauser, R. Hoop, P. Mattmann and S. Albini: Survey on Chlamydiaceae in cloacal swabs from Swiss turkeys demonstrates absence of Chlamydia psittaci and low occurrence of Chlamydia gallinacean. *PLOS ONE*, 14(12), e0226091 (2019) doi:10.1371/journal.pone.0226091

90. M. Taghavi, H. Ghorbani-Choboghlo, A. R. Khosravi, A. Erfanmanesh and A. Balal: Fungal flora of the combs and wattles of Iranian native chickens. *Iranian journal of microbiology*, 6(1), 46-50 (2014)

91. S. M. Hernandez-Divers, P. Villegas, C. Jimenez, S. J. Hernandez-Divers, M. Garcia, S. M. Riblet, C. R. Carroll, B. M. O'Connor, J. L. Webb and M. J. Yabsley: Backyard chicken flocks pose a disease risk for neotropic birds in Costa Rica. *Avian Diseases*, 52(4), 558-566 (2008)

92. M. A. Mohammed, S. M. Sokkar, A. Batrawi, M. K. Refaie and A. A. S. Ahmed: Natural and experimental nodular dermatomycosis in chickens. *Avian Pathology*, 9(2), 185-192 (1980) doi:10.1080/03079458008418402

93. P. K. Biswas, D. Biswas, S. Ahmed, A. Rahman and N. C. Debnath: A longitudinal study of the incidence of major endemic and epidemic diseases affecting semi-scavenging chickens reared under the Participatory Livestock Development Project areas in Bangladesh. *Avian Pathology*, 34(4), 303-312 (2005) doi:10.1080/03079450500178972

94. E. Cacciuttolo, G. Rossi, S. Nardoni, R. Legrottaglie and P. Mani: Anatomopathological aspects of avian aspergillosis. *Veterinary research communications*, 33(6), 521-527 (2009)

95. L. A. Beernaert, F. Pasmans, L. Van Waeyenberghe, F. Haesebrouck and A. Martel: Aspergillus infections in birds: a review. *Avian Pathology*, 39(5), 325-331 (2010) doi:10.1080/03079457.2010.506210

96. E. Beytut: Immunohistochemical diagnosis of aspergillosis in adult turkeys. *Turkish Journal of Veterinary and Animal Sciences*, 31(2), 99-104 (2007)

97. K. Jung, Y. Kim, H. Lee and J.-T. Kim: Aspergillus fumigatus infection in two wild Eurasian black vultures (Aegypius monachus Linnaeus) with carbofuran insecticide poisoning: A case report. *The Veterinary Journal*, 179(2), 307-312 (2009) doi:<https://doi.org/10.1016/j.tvjl.2007.09.004>

98. H. S. Ip, M. K. Torchetti, R. Crespo, P. Kohrs, P. DeBruyn, K. G. Mansfield, T. Baszler, L. Badcoe, B. Bodenstein, V. Shearn-Bochsler, M. L. Killian, J. C. Pedersen, N. Hines, T. Gidlewski, T. DeLiberto and J. M. Sleeman: Novel Eurasian highly pathogenic avian influenza A H5 viruses in wild birds, Washington, USA, 2014. *Emerging infectious diseases*, 21(5), 886 (2015) doi:10.3201/eid2105.142020

99. S. Nardoni, R. Ceccherelli, G. Rossi and F. Mancianti: Aspergillosis in Larus cachinnans micaellis: survey of eight cases. *Mycopathologia*, 161(5), 317-321 (2006)

100. J. Richard, T. Dvorak and P. Ross: Natural occurrence of gliotoxin in turkeys infected with Aspergillus fumigatus, Fresenius. *Mycopathologia*, 134(3), 167-170 (1996) doi:10.1007/BF00436725

101. E. A. Young, T. E. Cornish and S. E. Little: Concomitant Mycotic and Verminous Pneumonia in a Blue Jay from Georgia. *Journal of Wildlife Diseases*, 34(3), 625-628 (1998) doi:10.7589/0090-3558-34.3.625

102. E. Fagbohun, K. Ayantola and A. Toyin-Famoroti: Isolation and Molecular Characterization of Aspergillus fumigatus and Aspergillus flavus Isolated from Poultry Birds in Ado-Ekiti, Nigeria. *Asian Journal of Biotechnology and Bioresource Technology*, 31-44 (2020)

103. F. Ogasawara, Y. Yamamoto, Y. Sato, K. Fukunari, K. Murata, G. Yaegashi, M. Goto and R. Murakami: Concurrent Fowlpox and Candidiasis Diseases in Backyard Chickens with Unusual Pox Lesions in the Bursa of Fabricius. *Avian Diseases*, 60(3), 705-8 (2016) doi:10.1637/11397-022416-Case.1

104. J. D. Buck: Isolation of Candida albicans and halophilic Vibrio spp. from aquatic birds in Connecticut and Florida. *Applied and Environmental Microbiology*, 56(3), 826-828 (1990)

105. M. A. Mohammed, S. Sokkar, A. Batrawi, M. Refaie and A. Ahmed: Natural and experimental nodular dermatomycosis in chickens. *Avian Pathology*, 9(2), 185-192 (1980)

106. L. Bauck: Mycoses. In: *Avian Medicine: Principles and Applications*. Ed B. W. Ritchie, G. J. Harrison&L. R. Harrison. Wingers Publishing, Inc, Lake Worth, Florida (1994)

107. M. Lierz, A. Piepenbring, C. Herden, K. Oberhäuser, U. Heffels-Redmann and D. Enderlein: Vertical transmission of avian bornavirus in psittacines. *Emerging infectious diseases*, 17(12), 2390-2391 (2011) doi:10.3201/eid1712.111317

108. P. Delnatte, D. Ojkic, J. Delay, D. Campbell, G. Crawshaw and D. A. Smith: Pathology and diagnosis of avian bornavirus infection in wild Canada geese (Branta canadensis), trumpeter swans (Cygnus buccinator) and mute swans (Cygnus olor) in Canada: a retrospective study. *Avian pathology : journal of the W.V.P.A*, 42(2), 114-128 (2013) doi:10.1080/03079457.2013.769669

109. I. Preis, A. Fiúza, C. Silva, J. Braga, R. Couto, N. d. S. Martins and R. Ecco: Pathological, immunohistochemical, and molecular findings in commercial laying hens and in backyard chickens naturally infected with the infectious laryngotracheitis virus. *Brazilian Journal of Poultry Science*, 16, 359-366 (2014)

110. H. Hidalgo: Infectious laryngotracheitis: a review. *Brazilian Journal of Poultry Science*, 5, 157-168 (2003)

111. A. Tesfaye, M. Sahle, T. Sori, T. Kassa, A. Garoma, T. Koran, C. Dima, C. Guyassa, H. Hilu, S. Guta and F. Tadesse: Infectious Laryngotracheitis Virus in Commercial and Backyard Chicken Production Systems in Central and South Ethiopia (First report) ILT in Ethiopian Poultry Production. *Journal of Applied Poultry Research*, 28(4), 1324-1329 (2019) doi:<https://doi.org/10.3382/japr/pfz100>

112. K. Sary, S. Chénier, C. A. Gagnon, H. Shivaprasad, D. Sylvestre and M. Boulianne: Esophagitis and pharyngitis associated with avian infectious laryngotracheitis in backyard chickens: two cases. *Avian diseases*, 61(2), 255-260 (2017)

113. J. Blakey, S. Stoute, B. Crossley and A. Mete: Retrospective analysis of infectious laryngotracheitis in backyard chicken flocks in California, 2007-2017, and determination of strain origin by partial ICP4 sequencing. *Journal Of Veterinary Diagnostic Investigation*, 31(3), 350-358 (2019) doi:10.1177/1040638719843574

114. M. Islam, M. Khan, M. Islam and J. Hassan: Isolation and characterization of infectious laryngotracheitis virus in layer chickens. *Bangladesh Journal of Veterinary Medicine*, 8(2), 123-130 (2010)

115. P. Sun, S. Lin, S. He, E.-M. Zhou and Q. Zhao: Avian Hepatitis E Virus: With the Trend of Genotypes and Host Expansion. *Frontiers in Microbiology*, 10(1696) (2019) doi:10.3389/fmicb.2019.01696

116. X. Zhang, I. Bilic, S. Troxler and M. Hess: Evidence of genotypes 1 and 3 of avian hepatitis E virus in wild birds. *Virus Research*, 228, 75-78 (2017) doi:<https://doi.org/10.1016/j.virusres.2016.11.028>

117. R. Crespo, T. Opriessnig, F. Uzal and P. F. Gerber: Avian hepatitis E virus infection in organic layers. *Avian diseases*, 59(3), 388-393 (2015)

118. E. A. Turpin†, D. E. Stallknecht, R. D. Slemons, L. Zsak and D. E. Swayne: Evidence of avian metapneumovirus subtype C infection of wild birds in Georgia, South Carolina, Arkansas and Ohio, USA. *Avian Pathology*, 37(3), 343-351 (2008) doi:10.1080/03079450802068566

119. C. M. Jardine, E. J. Parmley, T. Buchanan, L. Nituch and D. Ojkic: Avian metapneumovirus subtype C in Wild Waterfowl in Ontario, Canada. *Transboundary and Emerging Diseases*, 65(4), 1098-1102 (2018) doi:10.1111/tbed.12832

120. L. S. Rizotto, R. M. Simão, G. P. Scagion, A. A. Simasaki, L. C. Caserta, J. C. Benassi, C. W. Arns and H. L. Ferreira: Detection of avian metapneumovirus subtype A from wild birds in the State of São Paulo, Brazil. *Pesquisa Veterinária Brasileira*, 39, 209-213 (2019)

121. T. Al-Shekaili, M. Baylis and K. Ganapathy: Molecular detection of infectious bronchitis and avian metapneumoviruses in Oman backyard poultry. *Research in Veterinary Science*, 99, 46-52 (2015) doi:<https://doi.org/10.1016/j.rvsc.2014.12.018>

122. I. Batista, P. Hoepers, M. Silva, P. Nunes, D. Diniz, A. Freitas, M. Cossi and B. Fonseca: Circulation of Major Respiratory Pathogens in Backyard Poultry and their Association with Clinical Disease and Biosecurity. *Brazilian Journal of Poultry Science*, 22 (2020)

123. A. Mussoyev, N. Assanov, G. Mussina, A. Sansyzbai and A. Valdovska: Serological aspects of avian metapneumovirus infection in Kazakhstan. *Research for rural development*, 1, 147 (2013)

124. M. Hristova and R. Petrova: COINFECTION OF CHICKEN ANAEMIA VIRUS, MYCOPLASMA GALLISEPTICUM, AVIAN METAPNEUMOVIRUS AND AVIAN REOVIRUS IN FANCY CHICKEN BREEDS. *TRADITION AND MODERNITY IN VETERINARY MEDICINE,*, 2(3), 17-22 (2017)

125. P. A. Felippe, L. H. A. d. Silva, M. B. d. Santos, S. T. Sakata and C. W. Arns: Detection of and phylogenetic studies with avian metapneumovirus recovered from feral pigeons and wild birds in Brazil. *Avian Pathology*, 40(5), 445-452 (2011) doi:10.1080/03079457.2011.596812

126. D. O. Oluwayelu, V. Smyth and D. Todd: Detection of avian nephritis virus and chicken astrovirus in Nigerian indigenous chickens. *African Journal of Biotechnology*, 11(17), 3949-3957 (2012)

127. T. Imada, S. Yamaguchi, M. Mase, K. Tsukamoto, M. Kubo and A. Morooka: Avian nephritis virus (ANV) as a new member of the family Astroviridae and construction of infectious ANV cDNA. *Journal of virology*, 74(18), 8487-8493 (2000) doi:10.1128/jvi.74.18.8487-8493.2000

128. W. Zhao, A. Zhu, Y. Yu, C. Yuan, C. Zhu, Z. Yang, L. Cui and X. Hua: Complete sequence and genetic characterization of pigeon avian nephritis virus, a member of the family Astroviridae. *Archives of virology*, 156(9), 1559-1565 (2011)

129. D. K. W. Chu, C. Y. H. Leung, H. K. K. Perera, E. M. Ng, M. Gilbert, P. H. Joyner, A. Grioni, G. Ades, Y. Guan, J. S. M. Peiris and L. L. M. Poon: A novel group of avian astroviruses in wild aquatic birds. *Journal of virology*, 86(24), 13772-13778 (2012) doi:10.1128/JVI.02105-12

130. P. Barrios, S. Marín, M. Resende, R. Rios, J. Resende, R. Horta, M. Costa and N. Martins: Occurrence of chicken anemia virus in backyard chickens of the metropolitan region of Belo Horizonte, Minas Gerais. *Brazilian Journal of Poultry Science*, 11, 135-138 (2009)

131. D. O. Oluwayelu and D. Todd: Rapid identification of chicken anemia virus in Nigerian backyard chickens by polymerase chain reaction combined with restriction endonuclease analysis. *African Journal of Biotechnology*, 7(3) (2008)

132. K. Hosokawa, K. Imai, H. V. Dong, H. Ogawa, M. Suzutou, S. H. Linn, A. Kurokawa and Y. Yamamoto: Pathological and virological analysis of concurrent disease of chicken anemia virus infection and infectious bronchitis in Japanese native chicks. *The Journal of veterinary medical science*, 82(4), 422-430 (2020) doi:10.1292/jvms.20-0006

133. D. A. Roussan: Serological survey on the prevalence of chicken infectious anemia virus in commercial broiler chicken flocks in Northern Jordan. *Int. J. Poult. Sci*, 5, 544-546 (2006)

134. B. Brener, R. Tortelly, R. C. Menezes, L. C. Muniz-Pereira and R. M. Pinto: Prevalence and pathology of the nematode Heterakis gallinarum, the trematode Paratanaisia bragai, and the protozoan Histomonas meleagridis in the turkey, Meleagris gallopavo. *Memorias do Instituto Oswaldo Cruz*, 101(6), 677-681 (2006)

135. A. M. MacDonald, C. M. Jardine, J. Bowman, L. Susta and N. M. Nemeth: Detection of lymphoproliferative disease virus in Canada in a survey for viruses in Ontario wild turkeys (Meleagris gallopavo). *Journal of wildlife diseases*, 55(1), 113-122 (2019) doi:10.7589/2018-01-013

136. J. M. Thomas, A. B. Allison, E. C. Holmes, J. E. Phillips, E. M. Bunting, M. J. Yabsley and J. D. Brown: Molecular Surveillance for Lymphoproliferative Disease Virus in Wild Turkeys (Meleagris gallopavo) from the Eastern United States. *PLOS ONE*, 10(4), e0122644 (2015) doi:10.1371/journal.pone.0122644

137. C. K. Stadler and J. W. Carpenter: Parasites of backyard game birds. *Seminars in Avian and Exotic Pet Medicine*, 5(2), 85-96 (1996) doi:<https://doi.org/10.1016/S1055-937X(96)80021-1>

138. T. Y. Morishita: Clinical Assessment of Gallinaceous Birds and Waterfowl in Backyard Flocks. *Veterinary Clinics of North America: Exotic Animal Practice*, 2(2), 383-404 (1999) doi:<https://doi.org/10.1016/S1094-9194(17)30129-9>

139. M. Rufai and A. Jato: Assessing the prevalence of gastrointestinal tract parasites of poultry and their environmental risk factors in poultry in Iwo, Osun state Nigeria. *Ife Journal of Science*, 19(1), 7-13 (2017)

140. C. Lagu and F. Kayanja: Medicinal plant extracts widely used in the control of Newcastle disease (NCD) and helminthosis among village chickens of South Western Uganda. *Livest Res Rural Dev*, 22 (2010)

141. J. R. Subedi, T. Mujahid and B. Cheetri: Prevalence of Intestinal Helminth Parasites of Chicken (Gallus gallus domesticus Linnaeus, 1758) in Lalitpur District, Nepal. *Tribhuvan University Journal*, 32(2), 19-28 (2018)

142. W. L. Wigle: Respiratory diseases of gallinaceous birds. *The veterinary clinics of North America. Exotic animal practice*, 3(2), 403-vi (2000) doi:10.1016/s1094-9194(17)30079-8

143. K. Wissler and O. Halvorsen: The Occurrence of gapeworm (Syngamus trachea) in willow grouse. *Journal of wildlife diseases*, 11(2), 245-247 (1975) doi:10.7589/0090-3558-11.2.245

144. G. L. Ivey and C. P. Herziger: Using Ivermectin to increase survival of Sandhill Crane colts at Malheur National Wildlife Refuge, Oregon. In: *Proceedings of the Eighth North American Crane Workshop*. Ed D. H. Ellis. North American Crane Working Group, Albuquerque, New Mexico (2001)

145. R. Katoch, A. Yadav, R. Godara, J. Khajuria, S. Borkataki and S. Sodhi: Prevalence and impact of gastrointestinal helminths on body weight gain in backyard chickens in subtropical and humid zone of Jammu, India. *Journal of Parasitic Diseases*, 36(1), 49-52 (2012) doi:10.1007/s12639-011-0090-z

146. M. H. Radfar, J. Khedri, K. Adinehbeigi, R. Nabavi and K. Rahmani: Prevalence of parasites and associated risk factors in domestic pigeons (Columba livia domestica) and free-range backyard chickens of Sistan region, east of Iran. *Journal of parasitic diseases*, 36(2), 220-225 (2012)

147. A. Permin, M. Bisgaard, F. Frandsen, M. Pearman, J. Kold and P. Nansen: Prevalence of gastrointestinal helminths in different poultry production systems. *British Poultry Science*, 40(4), 439-443 (1999) doi:10.1080/00071669987179

148. M. H. Radfar, S. Fathi, E. N. Asl, M. M. Dehaghi and H. R. Seghinsara: A survey of parasites of domestic pigeons (Columba livia domestica) in South Khorasan, Iran. *Veterinary Research*, 4(1), 18-23 (2011)

149. D. M. Tompkins, J. Greenman and P. J. Hudson: Differential impact of a shared nematode parasite on two gamebird hosts: implications for apparent competition. *Parasitology*, 122(2), 187-193 (2001)

150. E. E. Lund, A. M. Chute and G. C. Wilkins: The wild turkey as a host for Heterakis gallinarum and Histomonas meleagridis. *Journal of Wildlife Diseases*, 11(3), 376-381 (1975)

151. P. Bolfa, J. J. Callanan, J. Ketzis, S. Marchi, T. Cheng, H. Huynh, T. Lavinder, K. Boey, C. Hamilton and P. Kelly: Infections and pathology of free-roaming backyard chickens on St. Kitts, West Indies. *Journal of Veterinary Diagnostic Investigation*, 31(3), 343-349 (2019) doi:10.1177/1040638719843638

152. T. Ferdushy, M. T. Hasan and A. K. M. Golam Kadir: Cross sectional epidemiological investigation on the prevalence of gastrointestinal helminths in free range chickens in Narsingdi district, Bangladesh. *Journal of parasitic diseases : official organ of the Indian Society for Parasitology*, 40(3), 818-822 (2016) doi:10.1007/s12639-014-0585-5
